# Dissociated face- and word-selective intracerebral responses in the human ventral occipito-temporal cortex

**DOI:** 10.1007/s00429-021-02350-4

**Published:** 2021-08-09

**Authors:** Simen Hagen, Aliette Lochy, Corentin Jacques, Louis Maillard, Sophie Colnat-Coulbois, Jacques Jonas, Bruno Rossion

**Affiliations:** 1 CRAN UMR 7039, CNRS, Université de Lorraine, Pavillon Krug, Hôpital Central, CHRU-Nancy, 29 Avenue du Maréchal de Lattre de Tassigny, 54035 Nancy, France; 2 Donders Institute for Brain, Cognition and Behaviour, Radboud University, 6525 HR Nijmegen, The Netherlands; 3 Cognitive Science and Assessment Institute, University of Luxembourg, 365 Esch-sur-Alzette, Luxembourg; 4 Psychological Sciences Research Institute and Institute of Neuroscience, UCLouvain, 1348 Louvain-La-Neuve, Belgium; 5 Service de Neurologie, Université de Lorraine, CHRU-Nancy, 54000 Nancy, France; 6 Service de Neurochirurgie, Université de Lorraine, CHRU-Nancy, 54000 Nancy, France

**Keywords:** Face categorization, Word categorization, Frequency-tagging, SEEG, Fusiform gyrus, Anterior temporal lobe

## Abstract

The extent to which faces and written words share neural circuitry in the human brain is actively debated. Here, we compare face-selective and word-selective responses in a large group of patients (*N* = 37) implanted with intracerebral electrodes in the ventral occipito-temporal cortex (VOTC). Both face-selective (i.e., significantly different responses to faces vs. non-face visual objects) and word-selective (i.e., significantly different responses to words vs. pseudofonts) neural activity is isolated with frequency-tagging. Critically, this sensitive approach allows to objectively quantify category-selective neural responses and disentangle them from general visual responses. About 70% of significant electrode contacts show either face-selectivity or word-selectivity only, with the expected right and left hemispheric dominance, respectively. Spatial dissociations are also found within core regions of face and word processing, with a medio-lateral dissociation in the fusiform gyrus (FG) and surrounding sulci, respectively. In the 30% of overlapping face- and word-selective contacts across the VOTC or in the FG and surrounding sulci, between-category-selective amplitudes (faces vs. words) show no-to-weak correlations, despite strong correlations in both the within-category-selective amplitudes (face–face, word–word) and the general visual responses to words and faces. Overall, these observations support the view that category-selective circuitry for faces and written words is largely dissociated in the human adult VOTC.

**Supplementary Information:**

The online version contains supplementary material available at 10.1007/s00429-021-02350-4.

## Introduction

The human ventral occipito-temporal cortex (VOTC) is crucial for visual object recognition. While bilateral or right unilateral VOTC damage can cause a detrimental recognition impairment specific to faces (prosopagnosia, Bodamer 1947; see Bouvier and Engel 2006; Cohen et al. 2019 for lesion analysis), a selective lesion to the left VOTC can produce specific written word recognition impairment (pure alexia; Déjérine 1892; Cohen and Dehaene 2004). These neuropsychological findings of dissociated neural substrates associated with face *vs*. visual word recognition impairments (Farah 1991; Susilo et al. 2015; see also Robotham and Starrfelt 2017) have been complemented by neuroimaging evidence. Specifically, functional magnetic resonance magnetic (fMRI) studies have shown a larger neural response to faces than non-face objects in the lateral parts of the middle fusiform gyrus and in the inferior occipital gyrus with a right hemispheric dominance (e.g., Puce et al. 1995; Kanwisher et al. 1997; Rossion et al. 2012; see Grill-Spector et al. 2017 for review), while written words typically evoke larger responses than control stimuli in the left posterior fusiform and occipito-temporal sulcus (Devlin et al. 2006; Grill-Spector and Weiner 2014). While these findings support the view that the VOTC contains dissociated neural circuitry for face and written word recognition (Farah 1991), neuropsychological evidence of shared visual recognition impairments (Behrmann and Plaut 2014a, b; Rice et al. 2021) as well as fMRI studies showing partial spatial overlap between the functional face- and word-selective regions, in particular the fusiform gyrus (e.g., Davies-Thompson et al. 2016; Harris et al. 2015; Nestor et al. 2012), have probed researchers to propose instead that faces and words largely share the same high-level neural circuitry (Behrmann and Plaut 2013, 2020; Nestor et al. 2012).

Understanding the neural processes that generate recognition of visual words and faces is of high relevance for human social communication, because for most humans in literate societies, faces and written words constitute, arguably, the two most common, complex and socially relevant categories of their everyday visual environment. Thus, the claim that these two recognition functions are supported by shared neural circuits has implications for the large portion of the visual neuroscience community who studies either face or written word recognition. Importantly, this issue extends beyond the realm of faces and words, and speaks directly to how the brain is organized to perform stimulus–response mappings for highly experienced and behaviorally relevant visual categories in the two hemispheres, an issue cutting straight to the core of human neuroscience (Bradshaw and Nettleton 1981; Farah 1991; Behrmann and Plaut 2020).

To shed original light on this issue, we provide a comprehensive and systematic comparison of face and word category-selective neural responses with direct human intracranial electroencephalographic (iEEG) recordings. Previous iEEG studies have reported both face-selective (Allison et al. 1994, 1999; Halgren et al. 1994) and word-selective activity (Nobre et al. 1994; Thesen et al. 2012; Hirshorn et al. 2016) in the human VOTC, and two studies have compared these responses directly. In a brief report, Allison et al. (2002) showed intriguing opposite polarity responses of face- and word-evoked ERPs (N200/P200) on the same cortical sites in the VOTC. More recently, Matsuo et al. (2015) reported alternating zones selective to faces and written words in the VOTC in recordings restricted to six hemispheres (four individuals) where both multiple face- and letterstring-selective channels could be simultaneously identified in the VOTC. Thus, it remains unknown if the high co-occurrence of independently measured face- and word-selective responses in spatially confined regions (e.g., left FG) reflect shared or distinct neural circuitry.

Here, we take advantage of the recent extensive intracranial mapping of face-selective and word-selective responses in a common sample of individuals (Jonas et al. 2016; Lochy et al. 2018) to provide a direct comparison of the spatial overlap of these responses across the VOTC of both hemispheres. For a number of reasons, the present study goes well beyond the state-of-the-art concerning the spatial overlap/dissociation of neural substrates for face and written word recognition.

**Fig. 1:**
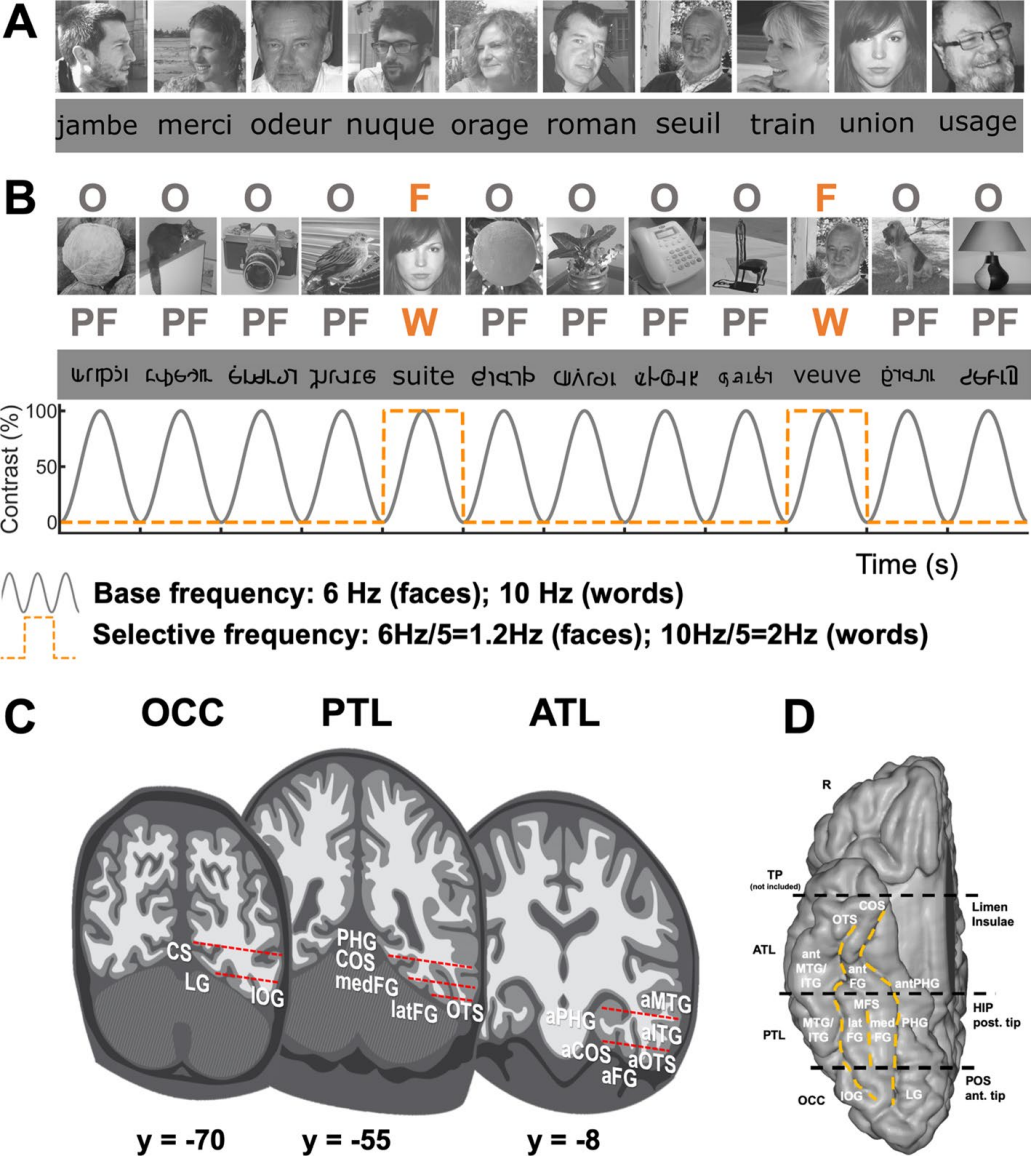
FPVS and SEEG methods. **A** Examples of stimuli for faces and words (actual face images not shown for copyright reasons). **B** For faces, images of living or non-living objects were presented by sinusoidal contrast modulation at a rate of six stimuli per second (6 Hz) with different images of faces presented in separate sequences every five stimuli (i.e., appearing at the frequency of 6 Hz/5 = 1.2 Hz). For words, pseudofonts (PF) were presented at a rate of ten stimuli per second (10 Hz) with different words presented in separate sequences every five stimuli (i.e., appearing at the frequency of 10 Hz/5 = 2 Hz). **C** Schematic coronal representation of the typical trajectories of depth electrodes implanted in the VOTC (adapted from Jonas et al. 2016; Lochy et al. 2018). Electrodes consist of 8–15 contiguous recording contacts (red rectangles) spread along the electrode length, along the medio-lateral axis. **D** Schematic representation of the parcellation scheme used to determine the anatomical label of each contact. Anatomical regions were defined in each individual hemisphere according to major anatomical landmarks. The ventral temporal sulci (COS, OTS, and midfusiform sulcus, i.e., MFS) serve as medial/lateral borders of regions, whereas two coronal reference planes containing anatomical landmarks (posterior tip of the hippocampus, i.e., HIP and anterior tip of the parieto-occipital sulcus, i.e., POS) serve as an anterior/posterior boundary for each region. We considered contacts in the ATL if they were located anteriorly to the posterior tip of the hippocampus. Note that we did not include in our analyses contacts in the temporal pole (TP), i.e., anterior to the limen insulae. The schematic locations of these anatomical structures are shown on a reconstructed cortical surface of the Colin27 brain. Acronyms: ATL: anterior temporal lobe; PTL: posterior temporal lobe; OCC: occipital lobe; PHG: parahippocampal gyrus; COS: collateral sulcus; FG: fusiform gyrus; ITG: inferior temporal gyrus; MTG: middle temporal gyrus; OTS: occipito-temporal sulcus; CS: calcarine sulcus; IOG: inferior occipital gyrus; LG: lingual gyrus; a: anterior; lat: lateral; med: medial

First, the electrophysiological recordings reported here are performed in a large sample of individual brains (*N* = 37; 61 implanted hemispheres) allowing extensive mapping of category-selective activity across the VOTC (Rossion et al. 2018). Second, rather than subdural grids of electrodes (Electrocorticography; ECoG; as used in Allison et al. 2002; Matsuo et al. 2015), depth electrodes, or intra*cerebral* recordings, are performed with StereoElectroEncephaloGraphy (SEEG, Talairach and Bancaud 1973). This is particularly important since SEEG samples neural activity inside the grey matter of both gyri and sulci, with a large portion of face- and visual word-selective activity in the posterior VOTC being disclosed in sulci (e.g., Occipito-temporal sulcus, collateral sulcus or mid-fusiform sulcus; see Grill-Spector et al. 2017; Grill-Spector and Weiner 2014 for reviews). Third, this approach provides direct measures of local cortical activity, allowing to explore up to the anterior sections of the VOTC, a region that is affected by large magnetic susceptibility artifacts in fMRI and has, therefore, been undersampled in terms of both face-selective and word-selective activity, not to mention their overlap, with this latter technique (Rossion et al. 2018; Wandell 2011). Finally, the study relies on a frequency-tagging approach, which provides an objective identification (i.e., at experimentally defined frequencies) and full quantification (as a sum of harmonics) of neural activity in the iEEG frequency domain while disentangling category-selective from general visual neural responses (see Norcia et al. 2015; Rossion et al. 2018). Specifically, the participants were tested here with two frequency-tagging experiments to isolate face- and word-selective responses. In the face condition, variable object images appear at a fixed frequency rate (*F*) with variable face images interleaved as every fifth item (*F*/5), while in the word condition, variable pseudofonts appear at a fixed frequency rate (*F*) with variable words interleaved as every fifth item (Fig. 1). As shown in scalp EEG and intracerebral recording studies, this approach objectively quantifies intracerebral face- and word-selective responses at the face- and word stimulation *F*/5 frequencies and harmonics (Rossion et al. 2015; Jonas et al. 2016; Lochy et al. 2018). Given that responses in the two conditions (face and word) are measured in the same set of electrode contacts in the same individual brains, including an extensive sampling of anterior VOTC regions, this approach is particularly well suited to test the issue of spatial and functional overlap of face- and word-selective neural activity in the VOTC. Importantly, the frequency-tagging paradigm allows for isolating category-*selective* responses at the *F*/5 frequency and parse out *general* visual responses at the base rate *F* frequencies (Rossion et al. 2018). Thus, if faces and words are represented by the same underlying neural circuits, one should observe largely overlap of *selective* responses obtained after parsing out shared general visual responses, as well as a high correlation of response amplitude across significant contacts for the two categories. In contrast, contacts responding to only faces or only words, as well as dissociations between response amplitudes within overlap contacts, would be consistent with the view that faces and words evoke category-selective activity in spatially distinct circuits.

## Methods

### Participants

The study included 37 patients (21 females, mean age: 32.9 ± 8.4 years, 36 right-handed) undergoing clinical intracerebral evaluation with depth electrodes (StereoElectroEncephaloGraphy, SEEG) for refractory partial epilepsy, studied in the Epilepsy Unit of the University Hospital of Nancy between September 2013 and June 2016. Patients were included in the study if they had at least one intracerebral electrode implanted in the VOTC (Fig. 1C). The SEEG data for word stimulation from 36 participating patients were included in Lochy et al. (2018), while the data for face stimulation from all patients were included in Hagen et al. (2020). All patients gave written consent to participate to the study, which was part of a protocol approved by the Ethics committee of the University Hospital of Nancy.

### Intracerebral electrode implantation and recording

Intracerebral electrodes were stereotactically implanted within the participants’ brains for clinical purposes, i.e., to delineate their seizure onset zones and to functionally map the surrounding cortex in the perspective of an eventual epilepsy surgery (Bédos-Ulvin et al. 2017). Each 0.8 mm diameter intracerebral electrode contains 8–15 independent recording contacts of 2 mm in length separated by 1.5 mm from edge to edge (for details about the electrode implantation procedure, see Salado et al. 2017). Intracerebral EEG was sampled at 512 Hz with a 256-channel amplifier and referenced to either a midline prefrontal scalp electrode (FPz, in 32 participants) or, when scalp electrodes were not available, an intracerebral contact in the white matter (6 participants). EEG signal was filtered during acquisition with a 0.15 Hz analog high-pass filter. Similar to previous intracranial reports, the data in subsequent analysis were not re-referenced (e.g., Allison et al. 2002; Hagen et al. 2020; Jonas et al. 2016; Kadipasaoglu et al. 2016; Lochy et al. 2018; Matsuo et al. 2015). However, a separate analysis using a bipolar re-reference yielded the same data pattern. Contacts located in brain lesions visible on structural MRI were excluded from any analysis**.** The recorded sequences were checked by an expert epileptologist (author JJ), and sequences with epileptic discharges or epileptic seizures were removed from the analysis.

### Fast periodic visual stimulation paradigm

#### Stimuli

In the face condition, we used 200 grayscale natural images of various non-face objects (from 14 non-face categories: cats [*n* = 9], dogs [*n* = 5], horses [*n* = 5], birds [*n* = 24], flowers [*n* = 15], fruits [*n* = 28], vegetables [*n* = 21], houseplants [*n* = 15], phones [*n* = 13], chairs [*n* = 15], cameras [*n* = 6], dishes [*n* = 15], guitars [*n* = 15], lamps [*n* = 14]) and 50 grayscale natural images of faces (see Fig. 1 for examples of stimuli; all images taken from a paradigm validated in scalp EEG studies, e.g., Rossion et al. 2015). Each image contained an unsegmented object or a face near the center. Faces and objects varied substantially in terms of size, viewpoint, lighting conditions and background across images (see Rossion et al. 2015). Images were equalized for mean pixel luminance and contrast (i.e., standard deviation across pixels) and resized to 200 × 200 pixels. Shown at a distance of 70 cm on a screen of size 300 mm height and 530 mm width and a resolution of 1280 × 720 pixels, the stimuli subtended approximately 6.72° of visual angle.

In the word condition, we used words and pseudofonts (30 of each type), all composed of 5 elements (letters or pseudofonts, PF) (Fig. 1A). These stimuli were also taken from a paradigm validated in scalp EEG studies (e.g., Lochy et al. 2015). French words were selected from the Lexique 3.55 database (New et al. 2001) with the following criteria: they were frequent common nouns (84.99 per million) in singular form, with limited orthographic neighbors (average 1.9; from 0 to 4), no foreign language origin, and no accents. PF items were built on an item-by-item basis: letters from words were vertically flipped, segmented, and segments were rearranged into five pseudoletters with the same overall size as the original word. Each word thus had a corresponding PF containing the exact same amount of black-on-white contrast, so that all conditions were similar in terms of lower level visual properties. Bigram frequencies were calculated with Wordgen (Duyck et al. 2004) and are reported as summated type bigram frequencies (from the French CELEX database). Stimuli were presented in Verdana font, with the size ranging from 4.8 to 7.7 (width) and 1.15 to 2 (height) degrees of visual angle.

#### Experimental procedure

The experiment was run using Sinstim, a custom Matlab software as in the original scalp EEG studies (Rossion et al. 2015; Lochy et al. 2015). In the face condition, participants viewed continuous sequences of natural images of objects presented at a fast rate of 6 Hz through sinusoidal contrast modulation, in which faces were presented periodically as every 5th stimulus so that the frequency of face presentation was 1.2 Hz (i.e., 6 Hz/5) (Fig. 1, Movie S1). All images were randomly selected from their respective categories. For the word condition, participants viewed continuous sequences of pseudofont strings (PF) presented periodically at a rate of 10 Hz through sinusoidal contrast modulation (from 0 to 100% in 50 ms, then back to 0% in 50 ms) with randomly selected words inserted every fifth item, so that the word presentation frequency was 2 Hz (10 Hz/5) (Fig. 1B and Movies S2). In all conditions, a sequence lasted 70 s: 66 s of stimulation at full-contrast flanked by 2 s of fade-in and fade-out, where contrast gradually increased or decreased, respectively. During the sequences, participants performed a color-change detection task on the fixation cross. In the face condition, they were instructed to detect brief (500 ms) black to red changes, while in the word condition, the fixation cross was blue and changed to red. In the face condition, sequences were repeated a minimum of two times (average sequences across patients: 2.67, 2–6). In the word condition, the experiment was repeated a minimum of two times for all but two patients (average sequences across patients: 2.92, 1–6). Note that different frequencies were used for faces (1.2, 6 Hz) and words (2, 10 Hz), because they were deemed highly sensitive for evoking category-selective responses in prior investigations examining the domains in isolation, thus maximizing the amount of overlap possibly detected (Rossion et al. 2015; Lochy et al. 2015).

#### Control procedure

In a control condition with houses, originally reported as part of a separate study on face and landmark dissociations in the VOTC (Hagen et al. 2020), participants viewed continuous sequences of natural images of objects presented periodically at 6 Hz through sinusoidal contrast modulation, with randomly selected house images inserted every fifth item, so that the frequency of house presentation was 1.2 Hz (i.e., 6 Hz/5) (see Fig. 1 in Hagen et al. 2020). Thus, the procedure was identical to faces with the exception that variable natural house images was interspersed at the fifth cycle rather than images of faces. Note that here we use the overlap between faces and houses strictly as a control comparison to face–word-overlap, and that a full report of face–house intracerebral responses, as measured with FPVS-SEEG, have been previously published (Hagen et al. 2020).

Participants were not informed about the periodicity of the stimulation and were unaware of the objectives of the study. No participant had seizures in the 2 h preceding fast periodic visual stimulation (FPVS) recordings.

#### Frequency domain processing

Signals corresponding to the faces﻿ and words conditions were processed the same way using the Letswave 5 toolbox for Matlab, as in our previous publications (Jonas et al. 2016; Lochy et al. 2018). Segments of SEEG corresponding to stimulation sequences were extracted (74-s segments, −2 s to + 72 s). The 74 s data segments were cropped to contain an integer number of 1.2 Hz cycles (for faces) and 2 Hz cycles (for words) beginning 2 s after the onset of the sequence (right at the end of the fade-in period) until approximately 65 s (for faces) and 66 s (for words), i.e., before stimulus fade-out (75 face cycles ≈ 63 s; 126 word cycles ≈ 63 s). No artifact correction was applied, because (S)EEG artifacts generate noise at frequencies that locate mostly outside of the frequencies of interest (1.2 or 2.0 Hz and associated harmonics) and, most importantly, the noise is broadband (*N* frequency bins noise: faces = 32,256; words = 32,768), while the signal locates in narrow frequency bins due to the very high-frequency resolution of our approach (*N* frequency bins signal: faces = 12; words = 4; Regan 1989; Rossion 2014). Thus, broadband noise with 1/*f* amplitude spectrum falls mainly outside the signal bins, and to further reduce the contribution of the 1/*f* noise our measure for amplitude quantification subtract the mean amplitude of the frequency bins surrounding each signal bin before summing the responses at different harmonics (description below). Nevertheless, to assess the effect of noise on the data, we performed the same quantification after artefact rejection, following the same procedure as previous reports (Jonas et al. 2016, appendix), yielding the same pattern of data as those without artifact rejection (Figure S1). Sequences of recorded voltage (i.e., time domain) were averaged separately for each participant and condition. Averaging sequences in the time domain before the Fast Fourier Transform (FFT) increases signal-to-noise ratio by cancelling out neural activity that is not phase locked to the stimulation (i.e., noise). Subsequently, an FFT was applied to the full length of the cropped averaged time sequences. The amplitude spectra were extracted for all contacts by taking the modulus of the Fourier coefficients at each frequency bin normalized (by dividing) by half of the number of time samples in the time series. The long recording sequence resulted in a spectrum with a high-frequency resolution of 0.0159 (1/63 s) for both faces and words (thus, faces and words did not differ in terms of frequency resolution). No data segments were excluded from the analysis. No other processing was performed to the data. The preprocessed data were subsequently analyzed in custom scripts in Matlab and Python.

#### Selective responses

**Fig. 2:**
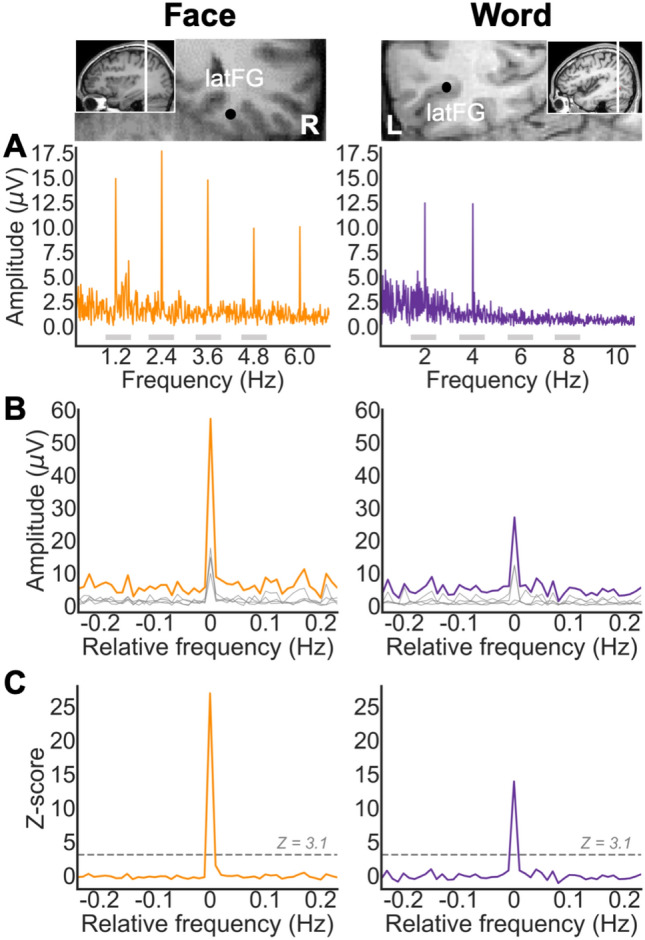
Intracerebral selective responses recorded in the VOTC. **A** Intracerebral EEG frequency-domain responses recorded at an individual recording contact (raw FFT amplitude) located in the right latFG of a single participant during a face stimulation sequence, and in the left latFG in a single participant during a word stimulation sequence. The anatomical location of the contact is shown in a coronal MRI slice. Face-selective responses are observed at 1.2 Hz and harmonics and word-selective responses are observed at 2 Hz and harmonics. **B** Significant face- and word-selective responses were determined by first segmenting the FFT spectrum into four segments centered at the frequency of face- and word- stimulation and its harmonics up to the 4th harmonic (i.e., faces: 1.2, 2.4, 3.6, and 4.8 Hz; words: 2, 4, 6, and 8 Hz). Individual FFT segments are shown in gray (see horizontal gray bars on the X axis in **A**, representing the length of each FFT segment). The four segments, containing both the signal and the surrounding noise, were then summed (orange and purple lines for faces and words, respectively). The 0 mark corresponds to the face or word stimulation frequencies. **C** *Z *score transformation of the summed FFT spectrum for statistical purpose. The *Z* score at the face/word frequency exceeds 3.1 (*p* < 0.001), indicating that these contact show significant face- or word-selective responses

**Fig. 3:**
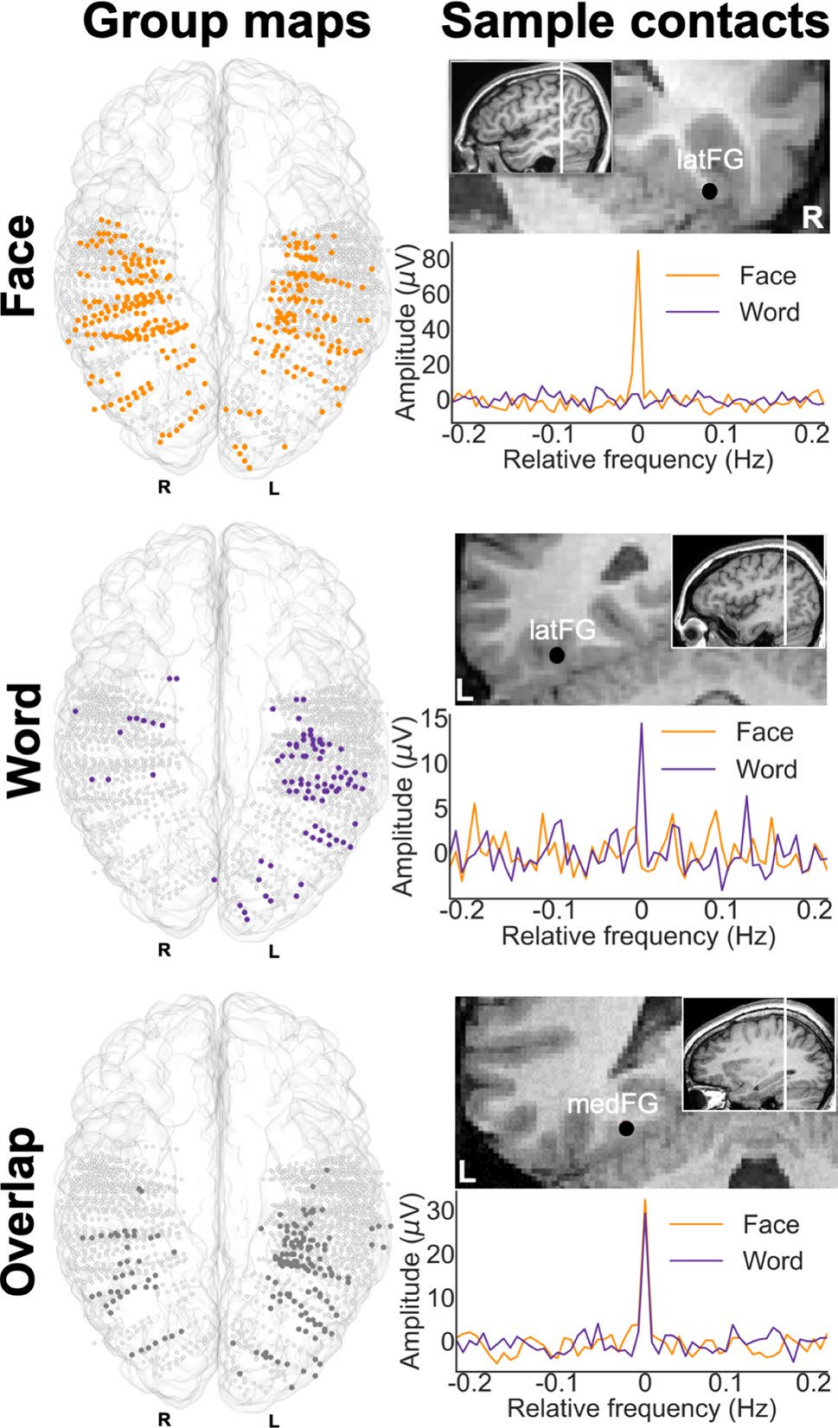
Classification and distribution of three types of category-selective contacts. **Left.** Maps of all 1969 VOTC recording contacts across the 37 individual brains displayed in the TAL space using a transparent reconstructed cortical surface of the Colin27 brain. Each circle represents a single contact. Color filled circles correspond to significant contacts colored according to their category selectivity (face, word, face–word-overlap). White-filled circles correspond to contacts on which no selective responses were recorded. **Right.** Examples of baseline corrected FFT spectra for each contact type (3 individual contacts in 3 different participants). Top: face contact, selective to faces only, middle: word contact selective to words only; bottom: face–word-overlap contact selective to both faces and words. Their anatomical location is illustrated in the respective coronal MRI slices

The FPVS approach used here allows identifying and separating two distinct types of responses in both conditions: (1) a general visual response occurring at the base stimulation frequency (faces: 6 Hz; words: 10 Hz) and its harmonics, as well as (2) a category-selective response at 1.2 Hz (faces) and 2 Hz (words) and its harmonics (face-selective or word-selective response, also called face categorization or word categorization response, respectively). In both face and word conditions, category-selective responses significantly above noise level at the face/word frequency (1.2 Hz/2 Hz) and its harmonics were determined as follows: (1) the FFT spectrum was cut into four segments centered at the face/word frequency and harmonics, from the first until the fourth (faces: 1.2 Hz until 4.8 Hz; words: 2 Hz until 8 Hz), and surrounded by 25 neighboring bins on each side (Fig. 2A); (2) the amplitude values in these four segments of FFT spectra were summed (Fig. 2B); (3) the summed FFT spectrum was transformed into a Z score (Fig. 2C). *Z* scores were computed as the difference between the amplitude at the face/word frequency bin and the mean amplitude of 48 surrounding bins (25 bins on each side, excluding the 2 bins directly adjacent to the bin of interest, i.e., 48 bins) divided by the standard deviation of amplitudes in the corresponding 48 surrounding bins. A contact was considered as showing a selective response in a given condition if the Z score at the frequency bin of face or word stimulation exceeded 3.1 (i.e., *p* < 0.001 one-tailed: signal > noise).

#### Classification of significant contacts

Based on the pattern of discrimination responses across the two conditions (i.e., significant or not), we labeled each significant contact as follows: (1) contacts showing a significant face-selective response, but not a significant word-selective response, were defined as “face” (+ face, −word); (2) contacts showing a significant word-selective response, but not a significant face-selective response, were defined as “word” (-face, + word); and (3) contacts showing significant selective responses to both faces and words, were defined as “overlap” (+ face, + word).

#### Quantification of response amplitude

Baseline-corrected amplitudes were computed as the difference between the amplitude at each frequency bin and the average of 48 corresponding surrounding bins (up to 25 bins on each side, i.e., 50 bins, excluding the 2 bins directly adjacent to the bin of interest, i.e., 48 bins). Face-selective responses were quantified separately as the sum of the baseline-subtracted amplitudes at the face frequency from the 1st until the 4th (1.2 Hz until 16.8 Hz), excluding the 5th and 10th harmonics (6 and 12 Hz) that coincided with the base rate frequency (Jonas et al. 2016). Word-selective responses were quantified separately as the sum of the baseline-subtracted amplitudes at the word frequency from the 1st until the 4th (2 Hz until 8 Hz; Lochy et al. 2018). The range of harmonics used was related to the highest harmonic with a significant response (Jonas et al. 2016; Lochy et al. 2018). Base rate response amplitudes were quantified separately as the sum of the baseline-subtracted amplitudes at the base frequency from the first until the fourth (faces: 6 Hz until 24 Hz; words: 10 Hz until 40 Hz), separately for faces and words sequences.

### Contact localization in the individual anatomy

The exact position of each contact in the individual anatomy was determined by fusing the postoperative CT scan with a T1-weighted MRI. Contacts inside the gray matter were anatomically labeled in the individual anatomy using the same topographic VOTC parcellation as in Lochy et al. (2018; Fig. 1D) based on anatomical landmarks. Major VOTC sulci (collateral sulcus and occipito-temporal sulcus) served as medio-lateral divisions. Postero-anterior divisions were the anterior tip of the parieto-occipital sulcus for the border between occipital and temporal lobes, and the posterior temporal lobe (PTL) and the anterior temporal lobe (ATL). In addition, we created an anatomical region-of-interest (ROI) consisting of the fusiform gyrus and surrounding sulci that are considered core to face and word processing (e.g., Kanwisher et al. 1997; Cohen et al. 2002; Harris et al. 2015; for review, see Grill-Spector and Weiner 2014). We refer to this region as FG + sulci, which according to our parcellation scheme (Fig. 1D), included latFG + OTS, medFG + COS, posterior antOTS, antFG, and posterior antCOS (Y Talairach < -25).

### Proportion and amplitude maps in Talairach space

In a separate analysis, anatomical MRIs were spatially normalized to determine Talairach coordinates of intracerebral contacts. Electrode locations were transformed to Talairach space by first locating the contacts in the original MRI system. Next, using Advanced Source Analysis (ASA, https://www.nitrc.org/projects/asa/), we determined three anatomical landmarks (nasion, left and right pre-auricular points) to define the fiducial system. Then, several points where determined in the MR volume (e.g., anterior and posterior commissure), to introduce the Talairach system, which was a piecewise linear transformation of the AC–PC system: anterior and posterior point (AP and PP; i.e. point of the cortex with maximum and minimum x coordinates), superior and inferior points (SP and IP; i.e. point of the cortex with maximum and minimum z coordinates), and right and left points (RP and LP; i.e. point of the cortex with maximum and minimum y coordinates) (Koessler et al. 2009, appendix). Talairach coordinates of the intracerebral contacts were used to perform group analyses and visualization. The cortical surface used to display group maps was obtained from segmentation of the Colin27 brain from AFNI (Cox 1996) which is aligned to the Talairach space. Using Talairach coordinates, we computed the local proportion and amplitudes of the discrimination intracerebral contacts across the VOTC. Local proportion and amplitudes of contacts were computed in volumes (i.e., “voxels”) of size 15 × 15 × 200 mm (for the X, left–right; Y, posterior–anterior; and Z, inferior–superior dimensions, respectively) by steps of 3 × 3 × 200 mm over the whole VOTC. A large voxel size in the Z dimension was used to collapse across contacts along the inferior–superior dimension. For each voxel, we extracted the following information across all participants in our sample: (1) the number of recorded contacts located within the voxel; (2) the number of contacts showing a significant response for each type of discrimination; and (3) the mean amplitudes in the significant contacts. For each voxel and each type of discrimination (i.e. face, word, overlap), we computed the proportion of significant contacts over recorded contacts (proportions are crucial here since sampling differs across regions), as well as the mean amplitudes over/in the significant contacts. To ensure reliability and reproducibility, we only considered voxels in which at least two participants showed significant responses. Then, for each voxel, we determined whether the proportion/amplitudes of significant contacts was significantly above zero using a bootstrap procedure in the following way: (i) sampling contacts from the voxel (the same number as the number of recorded contacts in the voxel) with replacement; (ii) determining the proportion of significant contacts for this bootstrap sample and storing this value; (iii) repeating steps i and ii 5000 times to generate a distribution of bootstrap proportions and to estimate the *p* value as the fraction of bootstrap proportions equal to zero.

### Correlation analysis

To compute within-category (e.g., face vs. face) and between-category (e.g., face vs. word) correlations, for each overlap-contact, two within-condition (e.g., face sequence 1 and 3) or between-condition sequences (face sequence 1 and word sequence 2) were randomly sampled, respectively. To account for different combinations of selections, this procedure was repeated 5000 times for each hemisphere, thereby creating 5000 sample correlations with associated values for *t*, *p*, and 95% confidence intervals (*CI*s). Finally, a grand average across the 5000 values (*r, t, p, 95%CIs*) was computed. Computing correlations based on sampling two sequences per intracerebral recording contact ensures that both the within- and between-category correlations are based on an equal amount of data (e.g., an alternative approach of splitting the within-category data would result in within-category correlations that was computed with half the amount of data compared to between-category correlations).

A permutation approach was used to statistically test correlations. First, to test for overall statistical significance (*R* > 0), (1) each of the 5000 sample correlations was recomputed and stored, after randomly shuffling the order of 1 data vector (to disrupt structure in the data), (2) this procedure was repeated 1000 times to produce a sampling distribution reflecting the null hypothesis, (3) estimate a *p *value as the fraction of permuted correlations larger than or equal to the real/measured correlation (two tailed). Second, to test for statistical difference relative to another correlation (e.g., R1 [face–face] vs. R2 [face–word] > 0), (1) each of the 5000 sample correlations for the 2 correlations (R1 and R2) was recomputed after combining, shuffling, and splitting half the data from each correlation (e.g., R1 [Face1–Face2] vs. R2 [Face3–Word1]: combine, shuffle, and split Face2 and Word1), (2) subtract and store the recomputed sample correlations to produce 5000 permuted difference correlations, (3) repeat this procedure 1000 times to produce a sampling null distribution, (4) estimate a P value as the fraction of permuted correlations larger than or equal to the difference of the real/measured correlations (one tailed).

## Results

### Visual face and word categorization

We found 566 contacts with category-selective responses (for faces, and/or words) in 36 individual brains, that is 28.75% of total recorded contacts (1969 contacts implanted in the grey matter of the VOTC in 37 subjects). Among these contacts, 15.90% were selective to words *only* (“word” contacts, 90/566, participants = 22), 53.36% were selective to faces *only* (“face” contacts, 302/566, participants = 33). Interestingly, 30.74% contacts were selective to both words and faces (“face-word-overlap” contacts, 174/566, participants = 24). In total, 16 out of 36 participants showed all three contact types, while the remaining participants showed the following combinations: face (*n* = 9); faces and words (*n* = 3); faces and overlap (*n* = 6); words and overlap (*n* = 2). Note that different contact types are expected across participants given that different participants have different electrode locations. An example of the response profile of each contact type is shown in Fig. 3. Each contact was localized in the individual anatomy using a topographic parcellation of the VOTC and in the Talairach space to perform group analyses and visualization (Fig. 3; see methods and Fig. 1D for the description of the parcellation; Table 1 for contact count by hemisphere and region).

**Table 1:** Face versus Words

Regions	Left hemisphere			Right hemisphere		
	Faces	Words	Overlap	Faces	Words	Overlap
VMO	14 (5)	11 (5)	17 (5)	20 (4)	0 (0)	4 (1)
IOG	6 (4)	8 (2)	19 (5)	12 (3)	0 (0)	9 (3)
Total OCC [133]	20 (9)	19 (7)	36 (10)	32 (7)	0 (0)	13 (4)
PHG	2 (1)	0 (0)	0 (0)	1 (1)	0 (0)	0 (0)
medFG	24 (10)	4 (3)	27 (12)	19 (6)	1 (1)	4 (2)
latFG	8 (5)	12 (6)	21 (8)	24 (6)	1 (1)	7 (3)
MTG/ITG	14 (6)	10 (6)	5 (3)	10 (4)	1 (1)	4 (1)
Total PTL [236]	48 (22)	26 (15)	53 (23)	54 (17)	3 (3)	15 (6)
antPHG	0 (0)	0 (0)	1 (1)	0 (0)	0 (0)	0 (0)
antCOS	29 (13)	17 (10)	8 (5)	25 (10)	6 (3)	3 (3)
antFG	2 (1)	0 (0)	10 (5)	13 (5)	0 (0)	3 (2)
antOTS	20 (11)	9 (7)	16 (9)	22 (9)	2 (2)	5 (2)
antMTG/ITG	9 (4)	7 (2)	7 (2)	28 (10)	1 (1)	4 (2)
Total ATL [817]	60 (29)	33 (19)	42 (22)	88 (34)	9 (6)	15 (9)

**Fig. 4:**
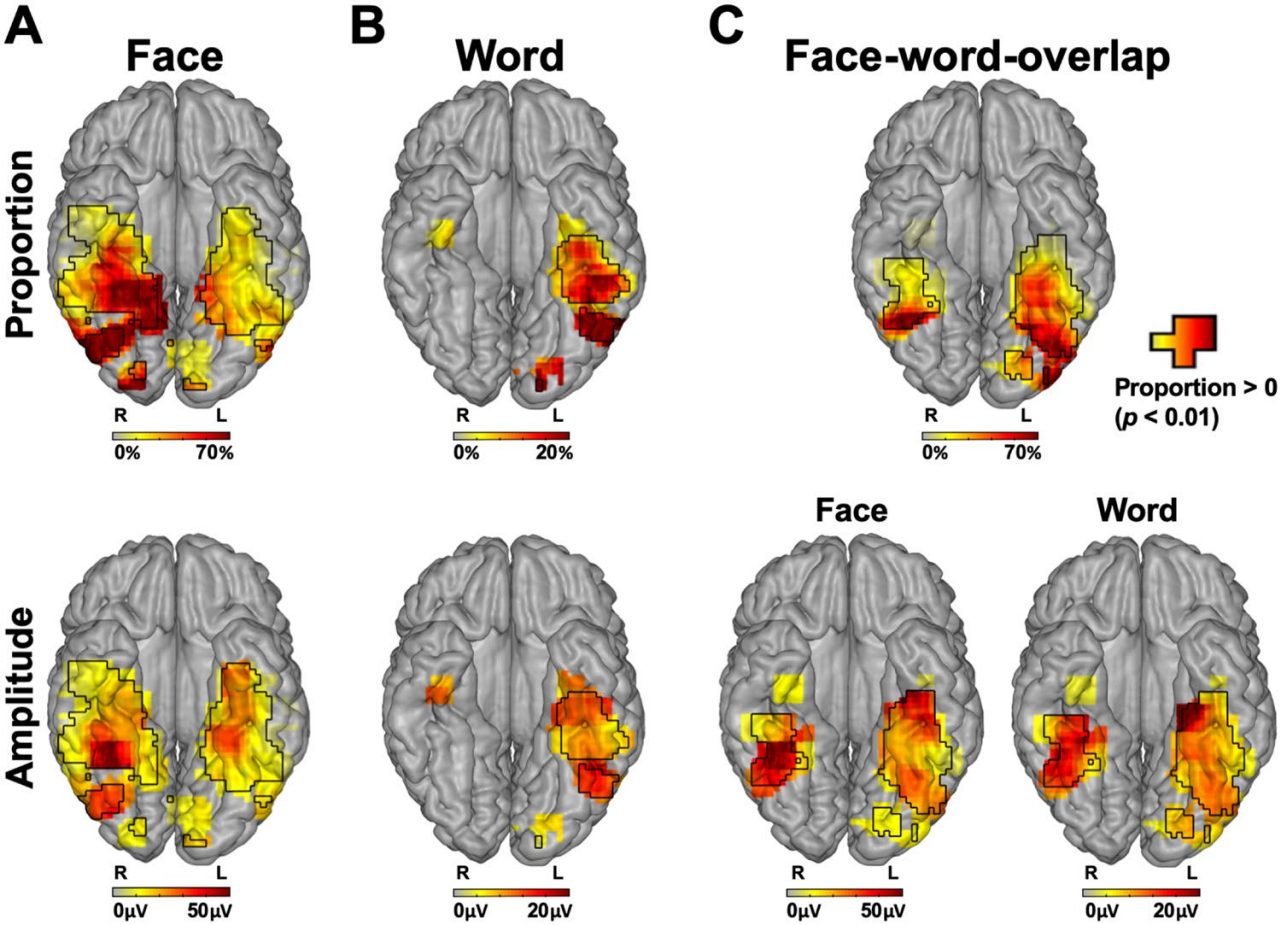
Proportion and amplitude maps. Top row: maps of the local proportion of selective contacts relative to recorded contacts across VOTC for each contact type (**A** face, **B** word, **C** overlap), displayed on the cortical surface. Bottom row: Maps of the local mean selective amplitudes for faces and words for each contact type (**A** face, **B** word, **C** overlap). Note that the overlap panel show both face-selective amplitudes and word-selective amplitudes within the overlap contacts. Local proportions and amplitudes were computed as total significant contacts divided by total recorded contacts and average amplitude across the significant contacts, respectively, in 15 × 15 voxels (for X and Y dimensions, respectively) using contacts collapsed over the Z dimension (superior–inferior) for better visualization. For the sake of replicability, only voxels containing significant responses from at least two individual brains were considered. Black contours outline proportions and amplitudes significantly above zero

Number of selective contacts and corresponding number of participants (in parentheses) in each anatomical region, and total recorded contacts in each larger region (in square brackets). See Fig. 4 for the number of significant contacts relative to total recorded contacts within local regions

*ATL* anterior temporal lobe, *PTL* posterior temporal lobe, *OCC* occipital lobe, *VMO* ventromedial occipital, *IOG* inferior occipital gyrus, *PHG* parahippocampal gyrus, *FG* fusiform gyrus, *MTG* middle temporal gyrus, *ITG* inferior temporal gyrus, *OTS* occipito-temporal sulcus, *COS* collateral sulcus, *ant* anterior, *lat* lateral, *med* medial

### Spatially and functionally dissociated face- and word-selective responses

To visualize and quantify face and word contacts at a group level, local proportions (out of total recorded contacts) and local average amplitudes (in significant contacts) were computed and projected on the cortical surface (Fig. 4). Contacts that were selective only to faces (face contacts) or only to words (word contacts) accounted for most of the total significant contacts (392/566 = 69.26%). Out of these contacts, there were more face than word contacts (diff = (302/566) (*F*) − (90/566) (W) = 37.46%; *χ2*(1, *N* = 566) = 175.39, *p* < 0.001). Moreover, the mean face-selective amplitude in face contacts (*M* = 19.02 μV) was larger than the mean word-selective amplitude in word contacts (*M* = 8.66 μV, *Mdiff* = 10.36 μV, *t(390)* = 5.69, *p* < 0.001).

**Fig. 5:**
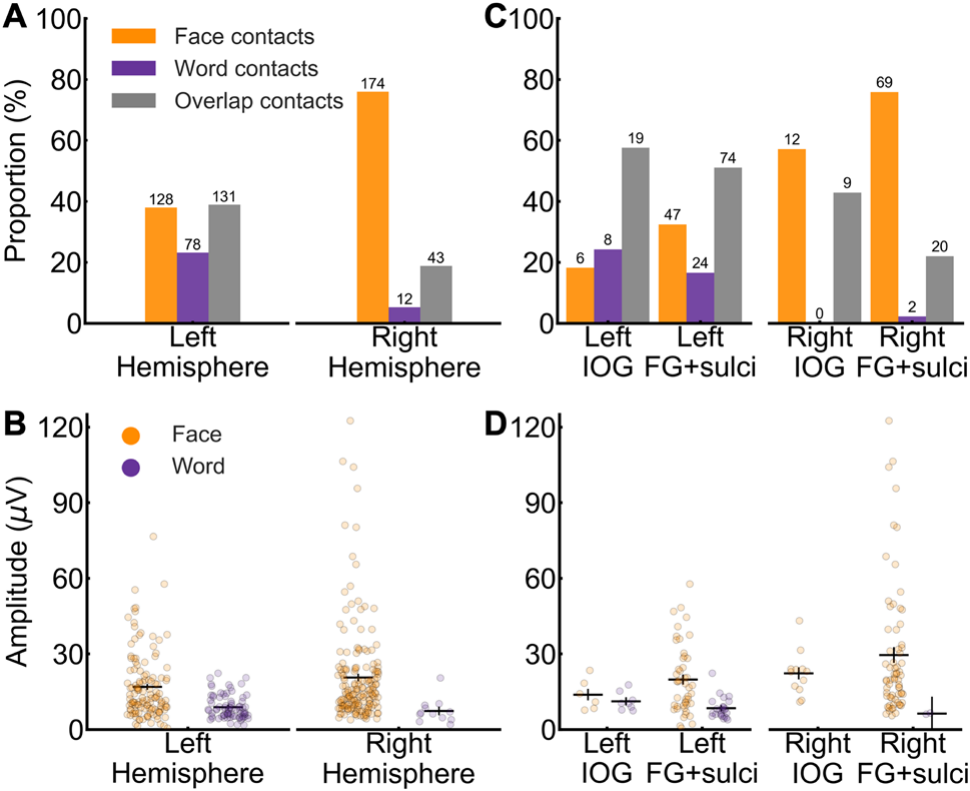
**A** Proportion of significant contacts (out of total significant contacts) split by contact type in each hemisphere. **B** Average selective amplitude in significant contacts split by contact type in each hemisphere. Each dot represents a single contact. **C** Proportion of significant contacts (out of total significant contacts) split by contact type in the IOG and FG + sulci. **D** Average selective amplitude in significant contacts split by contact type in the IOG and the FG + sulci. Same convention as panel B. The numbers on top of the bars in panel A and C indicate the number of significant contacts. Error bars in panels B and D represent standard error of the means

The face and word contacts showed the expected right and left hemispheric dominance, respectively. Specifically, faces made up a larger proportion of the significant contacts in the right than the left hemisphere (*diffhemi* = Right (R) – Left (L) = (174/229) − (128/337) = 38.00%, *χ2*(1, *N* = 566) = 79.11, *p* < 0.001; Fig. 5A), whereas the opposite was true for words (*diffhemi* = (12/229) (R) − (78/337) (L) = −17.91%, *χ2*(1, *N* = 566) = 32.69, *p* < 0.001; Fig. 5A). Moreover, for faces, there was a trend towards higher face-selective amplitudes in the right (*M* = 20.61 μV) than the left hemisphere (*M* = 16.87 μV; *Mdiff* = 3.74 μV, *t*(300) = 1.89, *p* = 0.060; Fig. 5B), while the low number of word contacts in the right hemisphere (Fig. 5A; *n* right hemisphere = 12) prevented the equivalent comparison for words (see Fig. 5B for point estimates).

The hemispheric dominance was also observed within anatomical regions thought to be core to face and word processing, the FG + sulci and IOG (e.g., Grill-Spector and Weiner 2014; Davies-Thompson et al. 2016), and where it has been proposed that faces and words compete for and share neural processes (Nestor et al. 2012; Behrmann and Plaut 2013). Specifically, in both the FG + sulci and the IOG, faces made up a larger proportion of significant contacts in the right than the left hemisphere (FG + sulci: *diffhemi* = (69/91) (R) − (47/145) (L) = 43.41%, *χ2*(1, *N* = 236) = 40.44, *p* < 0.001; IOG: *diffhemi* = (12/21) (R) − (6/33) (L) = 38.96%, *χ2*(1, *N* = 54) = 7.10, *p* = 0.008; Fig. 5C), whereas the opposite was true for words (FG + sulci: *diffhemi* = (2/91) (R) − (24/145) (L) = −14.35%, *χ2*(1, *N* = 236) = 10.33, *p* = 0.001; IOG: *diffhemi* = (0/21) (R) − (8/33) (L) = −24.24%, *χ2*(1, *N* = 54) = 4.21, *p* = 0.040; Fig. 5C). Similarly, in the FG + sulci and IOG, the face-selective amplitudes were larger in the right than the left hemisphere (*FG* + *sulci: Mdiff* = 29.50 μV (R) − 19.78 (L) = 9.72 μV, *t*(114) = 2.34, *p* = 0.021; IOG: *Mdiff* = 22.27 (R) − 13.82 (L) = 8.45 μV, *t*(16) = 2.10, *p* = 0.052; Fig. 5D; note low number of IOG contacts), while the low number of word contacts in the right hemisphere (IOG = 0; FG + sulci = 2) prevented the equivalent comparison for words (Fig. 5D).

**Fig. 6:**
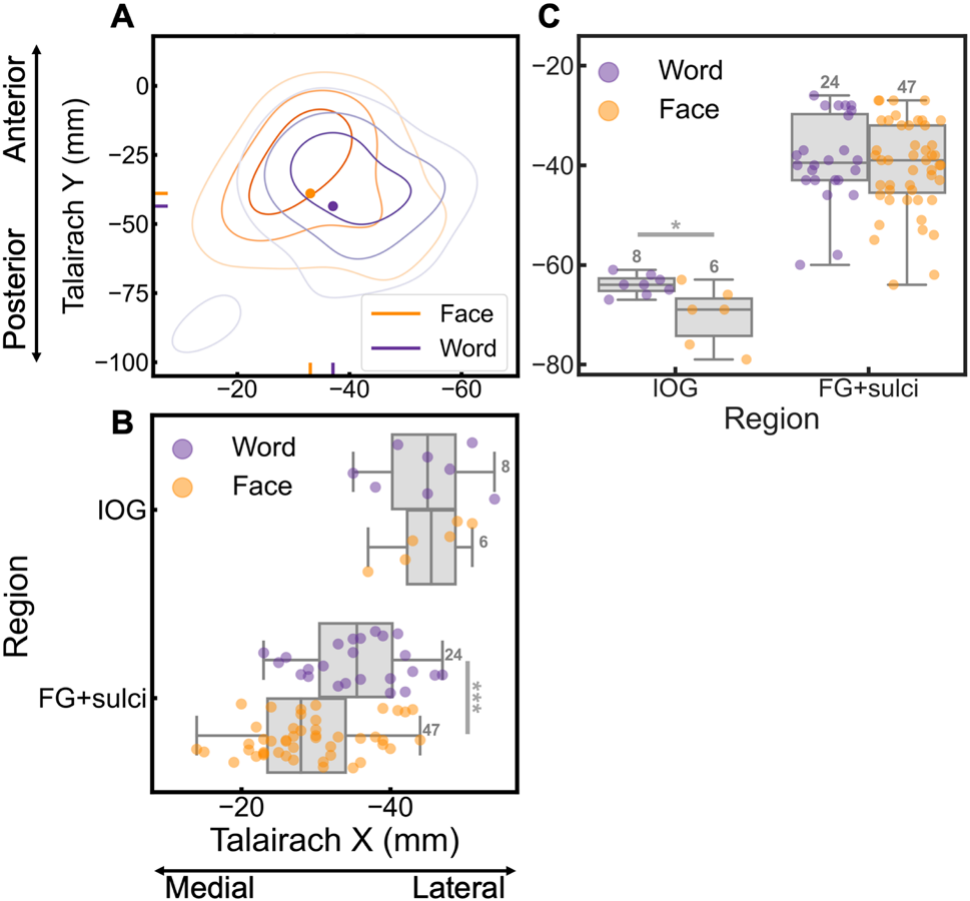
Spatial distribution of word and face contacts along medio-lateral (Talairach X) and posterio-anterior (Talairach Y) axis. The right hemisphere was not analyzed to due to it containing a low number of word contacts. **A** Contour plot showing distribution of word and face contacts in the left hemisphere along the medio-lateral (Talairach X) and posterio-anterior axes (Talairach Y). Each distribution is normalized relative to itself and darker contour colors indicate larger density of contacts. The central mass for each distribution is plotted as a solid dot within the contour plot and as a vertical/horizontal line on the x- and y-axis. **B** X coordinates of word and face contacts in core regions for neural processes supporting word and face recognition (IOG, FG + sulci). The boxplot displays three quartiles (Q1, median, Q2) and the whiskers extend to points that lie within 1.5 interquartile range (IQRs) of the lower and upper quartile. *p < 0.05; ***p < 0.001. The number next to the error bar indicate the number of contacts. Note that overlapping contacts are indicated by darker colors. **C** Y coordinates of word and face contacts in the IOG and FG + sulci. Same convention as for panel **B**

Next, we analyzed the differences between face and word contacts in their central mass along the X and Y Talairach axis, which was restricted to the left hemisphere due to the small number of significant word contacts in the right hemisphere (*n* = 12; see Fig. 4B first row; Table 1). Overall, the central mass of X Talairach coordinates (mean Talairach X) for word contacts was more lateral (*Mtalx* = 37.03 mm) than for the face contacts (*Mtalx* = 33.03 mm; *Mdiff* = 4 mm, *t*(df = 204) = 2.25, *p* = 0.026; Fig. 6A), while along the Y Talairach axis there was no difference between faces (*Mtaly* = −38.91 mm) and words (*Mtaly* = −43.51 mm; *Mdiff* = 4.60 mm, *t*(df = 204) = 1.42, *p* = 0.159; Fig. 6A). In the FG + sulci, there was also a medio-lateral dissociation (29.15 (F) − 35.46 (W) = −6.31 mm; *t*(*df* = 69) = −3.53, *p* < 0.001; Fig. 6B), but no posterior-anterior dissociation (−40.38 (F) − (−38.79) (W) = −1.59 mm; *t*(df = 69) = −0.71, *p* = 0.480; Fig. 6C). In contrast, in the IOG, there was no medio-lateral dissociation (45.00 (F) − 44. 63 (W) = 0.38 mm; *t*(*df* = 12) = 0.12, *p* = 0.909; Fig. 6B), but a posterior–anterior dissociation (−70.33 (F) − (−64.00) (W) = −6.33 mm; *t*(df = 12) = −2.80, *p* = 0.016; note few contacts in IOG; Fig. 6C), with face contacts located more posterior than word contacts. Thus, in addition to a large-scale dissociation along the medio-lateral axis, we found spatial dissociations within local regions that are core to face and word recognition.

In summary, more than two-thirds of all significant intracerebral contacts were functionally dissociated, either responding selectively only to faces or only to words. Moreover, these functionally dissociated contacts showed spatial dissociations, including opposite hemispheric dominance and dissociations along the medio-lateral and posterior–anterior axis.

### Overlapping but functionally dissociated face- and word-selective responses

Notably, a considerable proportion of contacts were also responding selectively to *both* faces and words (Figs. 3, 5A; Table 1). Does this overlap truly reflect shared neural populations recruited during both face and word processing?

**Fig. 7:**
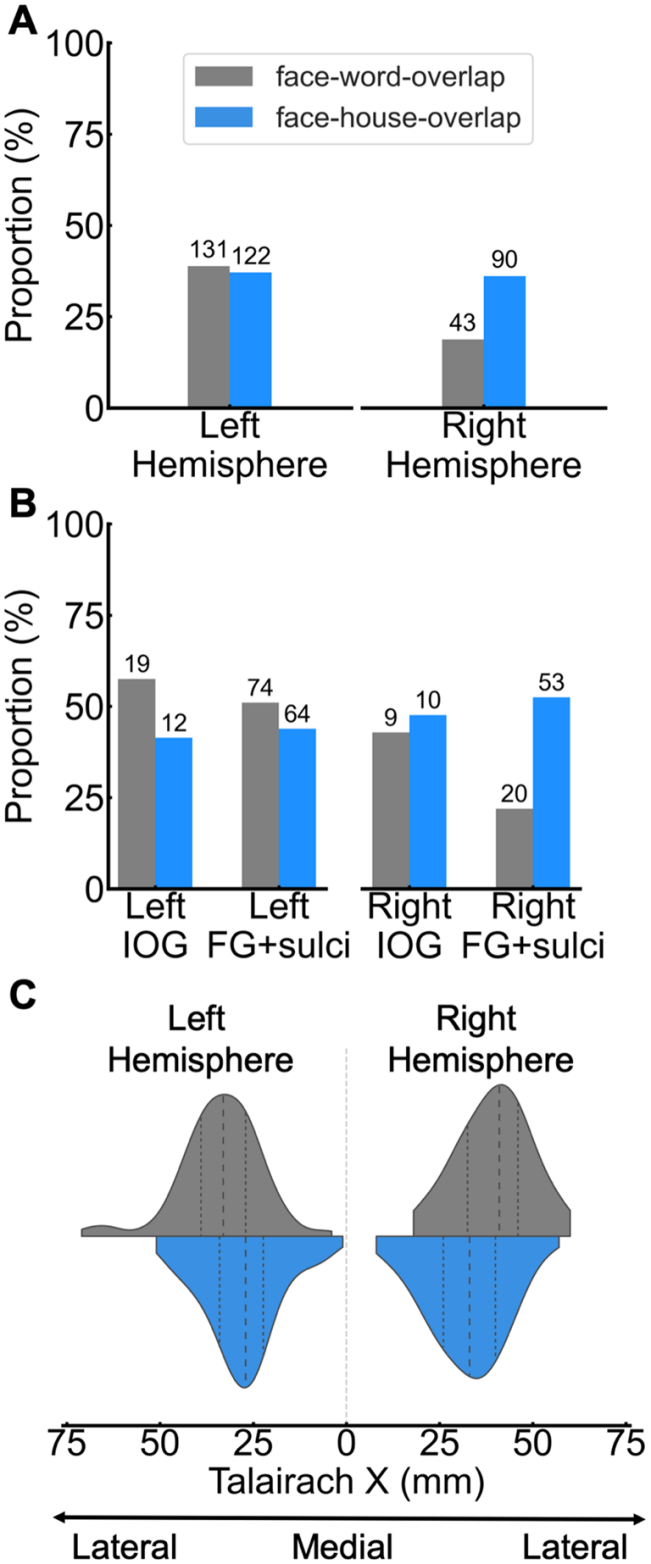
**A** Proportion of face–word-overlap (of total significant contacts [face, word, overlap]) and proportion of face–house-overlap contacts (of total significant contacts [face, house, overlap]), split by hemisphere. The proportion is expressed in percentage (%). The number of significant contacts is indicated on the top of each bar. **B** Proportion of overlap contacts, split by overlap-type, hemisphere and ROI. Same conventions as panel **A**. **C** Distribution of overlap contacts, split by overlap-type and hemisphere, along the Talairach X axis. Dashed lines within each distribution reflects the median and upper and lower quartiles. The range of each distribution is cut to fit the range of the observed data (i.e., the gaussian density estimation does not assign probability to contact coordinates lower or higher than observed data)

To answer this question, first, we examined the amount of face–word-overlap contacts in each hemisphere, the FG + sulci, and in the IOG. The face–word-overlap contacts accounted for a total of 30.74% (174/566) of all significant contacts and there was a larger proportion of overlap contacts (out of total significant contacts) in the left than in the right hemisphere (*diffhemi* = (43/229) (R) − (131/337) (L) = −20.10%, *χ2*(1, *N* = 566) = 25.86, *p* < 0.001*,* Fig. 7A). Most of the face–word-overlap contacts (122/174 = 70.12%) were found within anatomical regions that have been proposed to be central to face and word processes, the FG + sulci and the IOG (FG + sulci: 94/174 = 54.02%; IOG: 28/174 = 16.09%; FG + sulci + IOG = 70.11% of all face–word-overlap contacts). Within the FG + sulci there was a larger proportion (out of significant contacts) of face–word-overlap in the left than the right hemisphere (*diffhemi* = (20/91) (R) − (74/145) (L) = −29.06%, *χ2*(1, *N* = 236) = 18.50, *p* < 0.001; Fig. 7B), while there was an equal proportion in left and right IOG (*diffhemi* = (9/21) (R)—(19/33) (L) = −14.72%, *χ2*(1, *N* = 54) = −0.60, *p* = 0.438; Fig. 7B).

Next, motivated by the proposal that faces and words overlap due to their shared requirement for central-view visual representations (Plaut and Behrmann 2011), we examined overlap between faces and houses, since being landmark stimuli, pictures of houses are associated with spatially dissociated peripheral-view representations (e.g., Hasson et al. 2002; Levy et al. 2001; Malach et al. 2002), and show a medio-lateral spatial dissociation from faces (e.g., Grill-Spector and Weiner 2014). If central-view representation is a key factor driving the overlap between face- and word-selective responses, then we would expect more overlap between faces and words than faces and houses. House-selective responses were obtained in the same type of paradigm as for faces, by replacing faces with variable natural house images at the oddball rate of 1.2 Hz (see “Methods” for description of paradigm). Across all recorded contacts, contacts showing significant selective responses to both faces and houses were classified as face–house-overlap contacts (irrespective of how they responded to words). Note that here the overlap between faces and houses is used only as a reference point to assess the significance of the face–word-overlap and that a full description of face–house intracerebral responses, as measured with FPVS-SEEG, has been reported elsewhere (Hagen et al. 2020).

Notably, a substantial number of contacts also showed both face-selective and house-selective responses (212/578 = 36.68% of significant face and house contacts). There was an equal proportion (out of significant face, house, face-house-overlap contacts) of overlap across hemispheres (*diffhemi* = (90/249) (R) − (122/329) (L) = −0.94%, *χ2*(1, *N* = 578) = 0.05, *p* = 0.817*,* Fig. 7A). Crucially, a substantial portion of these were also located within the IOG and the FG + sulci (FG + sulci: 117/212 = 55.19%; IOG: 22/212 = 10.38%; FG + sulci + IOG = 65.57% of all face–house-overlap contacts). There was an equal proportion of face–house-overlap contacts in the right and the left hemispheres of both the FG + sulci and the IOG (FG + sulci: *diffhemi* = (53/101 (R) − (64/146) (L) = 8.64%, *χ2*(1, *N* = 247) = 1.79, *p* = 0.181; IOG: *diffhemi* = (10/21) (R) − (12/29) (L) = 6.24%, *χ2*(1, *N* = 50) = 0.19, *p* = 0.661; Fig. 7B). Thus, the spatial overlap between selective responses to faces and selective responses to words was not outstanding, since a similar amount of overlap was observed between faces and houses, both within hemispheres as well as within core face- and word-regions.

Next, we analyzed the face–word-overlap and face–house-overlap contacts along the X Talairach axis. One possibility is that the neural sources generating face, word and house responses are spatially distinct, yet are measured on the same recording contacts due to their proximity to each other. If this is the case, then the centre of mass of face–word-overlap contacts should be situated more laterally than the face–house-overlap contacts, since word-selective and house-selective responses are localized more laterally and medially, respectively, to face-selective responses (e.g., fMRI: Grill-Spector and Weiner 2014; Hasson et al. 2002; Spiridon et al. 2006; Nasr et al. 2011; intracranial EEG: Hagen et al. 2020; Kadipasaoglu et al. 2016; Jacques et al. 2016). Consistent with this claim, in both hemispheres, the centre of mass of the face–word-overlap contacts (Right: *Mtalx* = 39.54 mm; Left: *Mtalx* = 33.83 mm) was more lateral than that of the face–house-overlap contacts (Left: *Mtalxdiff* = 5.96 mm, *t*(251) = 4.51, *p* < 0.001; Right: *Mtalxdiff* = 7.11 mm, *t*(131) = 3.78, *p* < 0.001; Fig. 7C).

**Fig. 8:**
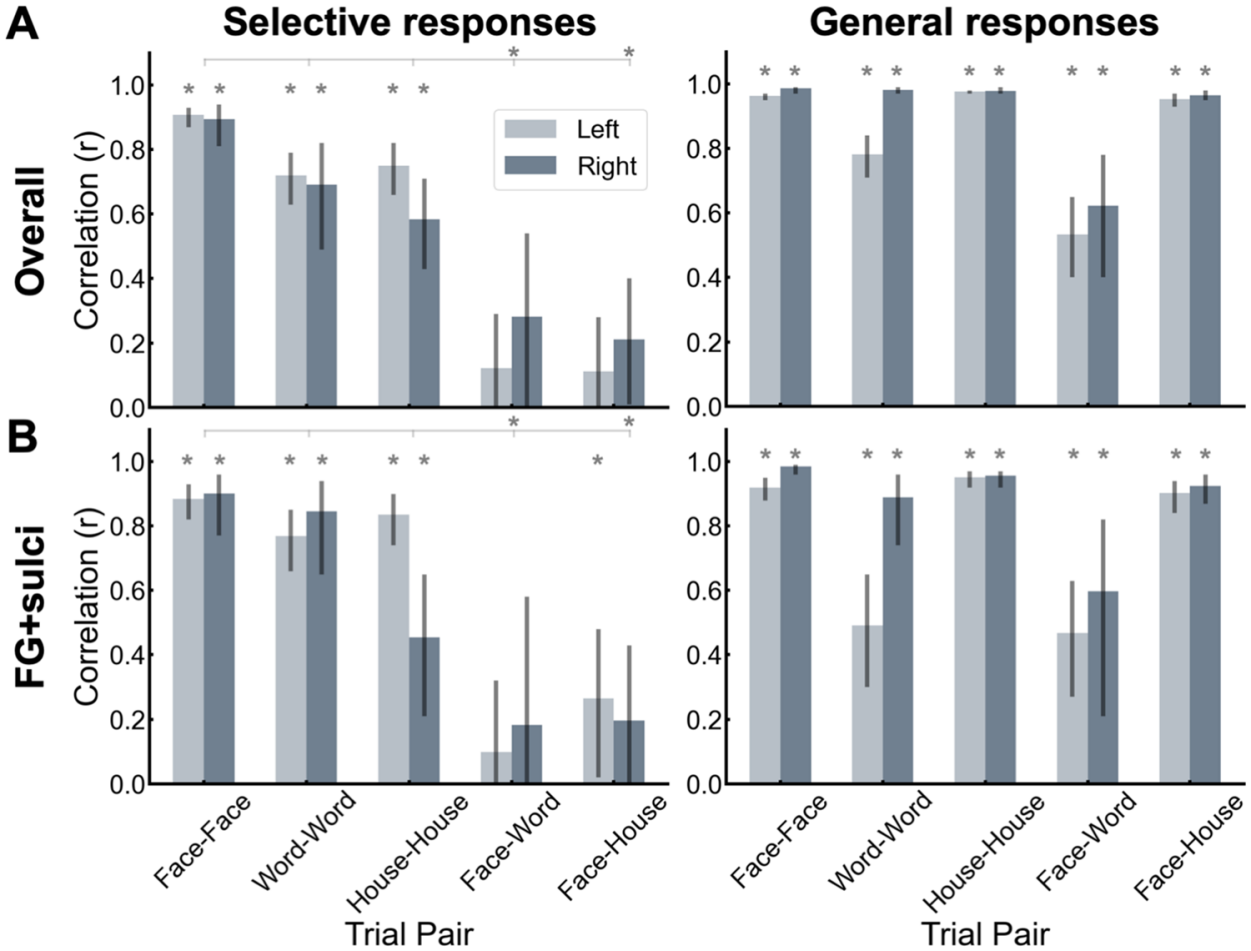
Correlation between the face-, word- and house-selective response amplitudes within overlap contacts. **A** Correlations across all overlap contacts, split by hemispheres, for (left) selective discrimination responses and (right) general visual responses. **B** Correlations across all overlap contacts in the FG + sulci, split by hemispheres, for (left) selective discrimination responses and (right) general visual responses. *r ≠ 0; Error bars represent 95% CIs. FG: fusiform gyrus

To directly test the extent of dissociation in the selective responses in the overlap contacts, we correlated face- and word-selective response amplitudes in face–word-overlap contacts, as well as the face- and house-selective responses in the face–house-overlap contacts. A shared neural population account would predict strong correlations between faces and words (which should be larger than that of faces and houses) since they are reflecting the same neural population. This was tested by correlating between-category response amplitudes across overlap contacts (i.e., faces vs. words; faces vs. houses), and comparing them to within-category correlations (i.e., faces vs. faces, words vs. words, houses vs. houses) in the same contacts. This was done separately by hemispheres (Fig. 8A) and FG + sulci (Fig. 8B), except in the IOG due to its low number of significant overlap contacts (face–words-overlap: *n* right = 9; *n* left = 19; face–houses-overlap: *n* right = 12; *n* left = 10).

As shown in Fig. 8A, in the face–word-overlap contacts, correlation between the face- and word-selective amplitudes in the left and right hemispheres was not significantly higher than 0, despite near-ceiling correlations for within-category amplitudes in both hemispheres. Moreover, a similar pattern was observed for faces and houses in face–house-overlap contacts (see “Methods” for description of correlation analysis and statistics; see figure below and SI for stats).

In contrast to the selective discrimination responses, for the general visual responses for both the face–word-overlap and the face–house-overlap contacts, there were strong within-condition (e.g., face–face) and between-condition (e.g., face–word) correlations in both hemispheres. This suggest that the lack of correlations in the selective *discrimination* responses cannot be attributed to different levels of noise or attention across conditions, since both the general visual responses and the discrimination responses were measured concurrently within each contact (Fig. 8A; see figure and SI for stats). Finally, similar patterns were observed when considering electrode contacts only within the FG + sulci, both for the discrimination responses and the general visual responses (Fig. 8B; see figure below and SI for stats).

In summary, there was no-to-weak correlations of between-category responses (e.g., face–word), both at the hemispheric-level and within FG + sulci, despite the same contacts showing strong within-category (e.g., face–face, word–word) correlations. Moreover, contrary to the selective responses, the general responses showed both strong within- and between-category correlations, showing that the lack of between-category correlation in the selective responses is not due to lack of attention, disproportionate noise level, or number of cycles in the respective domains. Separate control analysis showed that the lack of between-category correlations (e.g., face–word), both overall and in the FG + sulci, remained unchanged when using the same number of harmonics summed for faces and words (both 4 and 12 harmonics) or the same frequency range for the selection of harmonics (until 12 Hz for both conditions; Figure S2). Notably, the same patterns of correlations were found for face–house-overlap contacts. Overall, these observations speak against the claim of a special relationship between faces and words in recruiting overlapping category-selective neural processes.

**Fig. 9:**
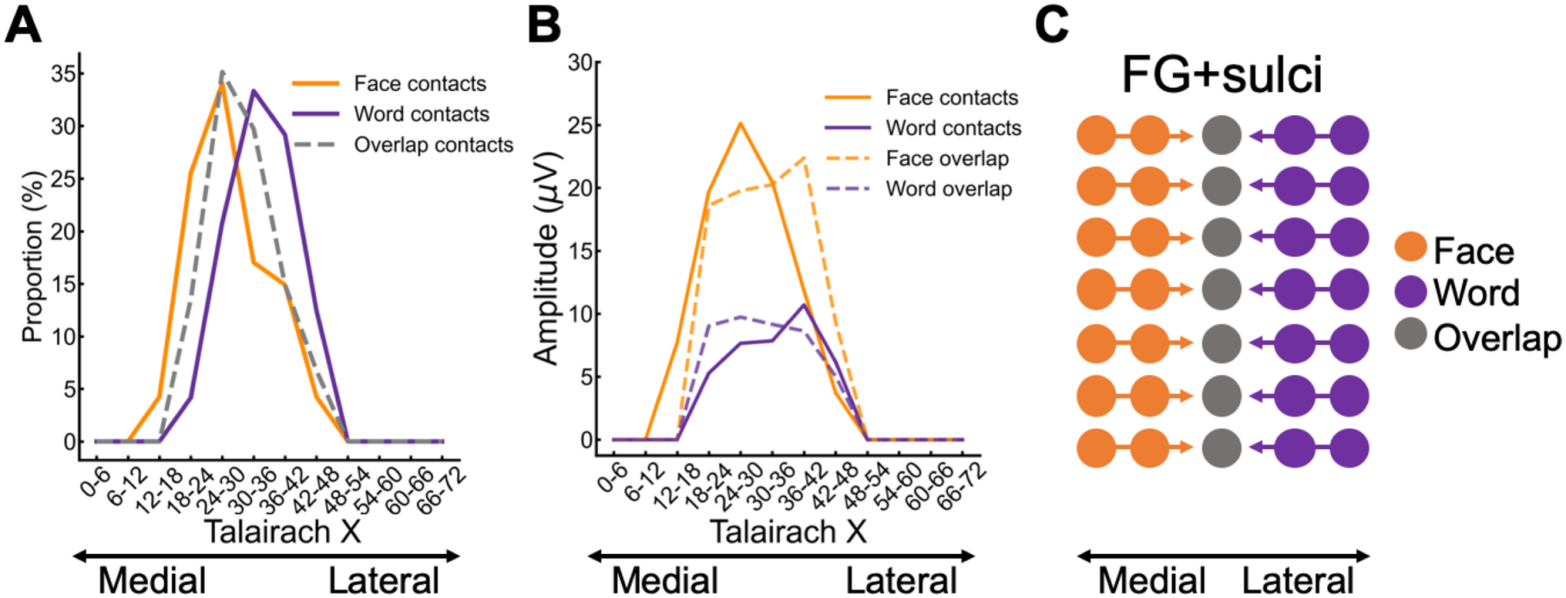
**A** Spatial position of face–word-overlap contacts relative to face and word contacts on the Talairach X axis. **B** Average selective response amplitudes to faces and words as a function of X Talairach in face and word contacts (solid lines) and overlap contacts (dashed lines). **C** Schematic of the hypothesized neural sources of the responses recorded on the overlap contacts

A final analysis examined if the face- and word-selective amplitudes within the face–word-overlap contacts showed a spatial dissociation along the medio-lateral axis (X Talairach). We included similar analysis for face and word contacts for comparison, and we restricted analysis to the left FG + sulci, since this region showed a spatial dissociation between face and word contacts and due to the low number of contacts in that hemisphere (Fig. 6). First, we observed that the overlap contacts were located in between face and word contacts on the X Talairach axis (Fig. 9A). Second, to compare amplitudes along X Talairach, we computed the mean selective response amplitude within 12 equally spaced X Talairach bin (size of 6 mm) separately for face, word, and overlap contacts in the left FG + sulci (Fig. 9B). For each contact type, we generated a distribution of Talairach coordinates, with coordinates corresponding with the center of the X Talairach bin, proportional to the mean selective amplitude in each bin (e.g., amplitudes 3 and 6 yielded 3 and 6 coordinate data points, respectively). Thus, the mode in each distribution reflects the X Talairach coordinate with the highest amplitudes, unlike the mode for the count data, which reflects the X Talairach coordinate with the highest density of contacts. To deal with potential amplitude outliers in each bin, we winsorized the amplitude distribution across contacts separately for each contact type (limits = 0.1, 0.9). Direct statistical comparison of the face and word contacts revealed that the face-selective amplitudes (*Mtalx* = 28.35 mm) centered more medially than did the word-selective amplitudes (Fig. 9B; *Mtalx* = 33.79 mm; diffFG + sulci = 5.44, *t* =  − 3.58, *p* < 0.001). In contrast, the overlap contacts, showed no difference in their center of mass for face-selective (*Mtalx* = 31.8 mm) and word-selective amplitudes (Fig. 9B; *Mtalx* = 31.71 mm; *Mdiff* = 0.09 mm, *t* = 0.06, *p* = 0.954). However, the face and word amplitude *magnitudes* in the face-word-overlap contacts mirrored the corresponding amplitudes in the face and word contacts, respectively.

Overall, the lack of correlation and dissociation in amplitude magnitudes in face- and word-selective amplitudes, suggest that the face-word-overlap contacts in left FG + sulci record from dissociated face- and word-selective neuronal groups that project to the same intracerebral contacts (Fig. 9C). If functionally different neuronal groups are proximally situated—as indicated by the dissociated face and word contacts in the FG + sulci (Fig. 6; Fig. 9A)—it is not unexpected that *LFPs* of intracerebral contacts reflect responses from both groups.

## Discussion

We examined the degree to which faces and written words evoke overlapping or dissociated neural responses in the human VOTC, using a large-scale intracerebral recording approach. With two highly sensitive frequency-tagging paradigms to objectively evoke and quantify category-selective responses in the respective domains, thereby increasing the ability to detect overlap, we found that more than two-thirds of intracerebral contacts responded selectively either *only* to faces (i.e., vs. non-face objects) or only to written words (i.e., vs*.* pseudofonts). These contacts showed strong responses in FG + sulci, with a right and left lateralization for faces and words, respectively, and were dissociated along the medio-lateral axis in the FG + sulci, and the posterior–anterior axis in the IOG. While the 30% of contacts at which selective responses to *both* faces and words were found could be taken as evidence of an overlap in neural circuitry, a similar amount of overlap was found between faces and a control category, houses. Crucially, there was little-to-no correlation in response amplitude between the face- and word-selective responses in face–word-overlap contacts, either within hemispheres or core regions of face- and word-processing (i.e., FG + sulci). The lack of significant correlations between faces and words cannot be explained by differences in presentation frequency, attentional state, or noise, since the concurrently measured *general* visual responses showed a strong correlation, despite vastly different base stimuli (objects and pseudofonts). Finally, in the left FG + sulci, the magnitude of face- and word-selective response amplitudes in the overlap contacts were dissociated, and mirrored those of flanking medial face and lateral word contacts, respectively. Overall, these observations indicate that category-*selective* human intracerebral responses to faces and written words reflect spatially proximal but distinct neural circuitry in the human VOTC.

FMRI studies consistently find, especially in fusiform gyrus and surrounding sulci, substantially larger responses to faces (FFA) and words (VWFA) relative to control stimuli (e.g., Puce et al. 1995; Kanwisher et al. 1997; Cohen et al. 2000; Cohen and Dehaene 2004; Baker et al. 2007; Davies-Thompson et al. 2016), and these functionally defined areas partially overlap in the FG + sulci (e.g., Harris et al. 2015). Similarly, ECoG studies have reported both distinct and overlapping responses to faces and written words, with a bias for overlap towards the FG (Matsuo et al. 2015). The functional relevance of this overlap is contentious as it has been proposed to reflect largely shared neural circuitry (Behrmann and Plaut 2013, 2020) or distinct but spatially overlapping neural circuitry (e.g., Harris et al. 2015). While fMRI studies have examined the functional relationship between the neural patterns in response to the presentation of faces and written words in VOTC regions sensitive to words (Nestor et al. 2012) or defined anatomically (Harris et al. 2015), no study has isolated and compared the category-*selective* responses to rule out the contribution of confounding general visual responses (i.e., faces > control vs. words > control) and there is, to our knowledge, no iEEG data to complement the (few) fMRI studies on this topic. The current study fills this gap of knowledge by using two frequency-tagging paradigms developed to isolate face- and word-selective responses in electrophysiology (Rossion et al. 2015; Lochy et al. 2015), coupled with direct neural recordings from spatially sensitive intracerebral-depth-electrodes implanted in the grey matter of both sulci and gyri in a large group of patients. Thus, this approach offers a unique opportunity to examine the claim that faces and words are represented in high-level visual cortex in a shared distributed system whereby both domains are represented in the same units in a graded fashion (Behrmann and Plaut 2020). According to this view, faces and words should overlap at each contact since they are represented in the same underlying “units”. However, the little overlap observed and the dissociations in the response amplitudes within these contacts, argues against this view. Therefore, we suggest that the dissociations observed are more in line with the view that faces and words rely on entirely distinct neural circuits in the VOTC (Farah 1991; Puce et al. 1996; Dehaene-Lambertz et al. 2018).

Strong evidence supporting the view that face and word recognition rely on spatially close yet dissociated neural circuitry comes from neuropsychological studies: while shared visual recognition impairments for faces and written words may be found in cases of general visual object agnosia (Farah 1991; Behrmann and Plaut 2014a, b) or in large cohorts of patients defined based on posterior brain lesions (Rice et al. 2021), brain-damaged patients can show highly specific impairments in the recognition of faces (prosopagnosia) or written words (pure alexia). These impairments typically occur as a result of a brain injury in the vicinity of the right and left, respectively, FG and inferior occipital gyrus (IOG) (Farah 1991; Farah et al. 1998; Behrmann and Bub 1992; Gaillard et al. 2006; Susilo et al. 2015; Cohen et al. 2019; see also, Robotham and Starrfelt 2017). Moreover, neuroimaging studies consistently report enhanced face-selectivity in the right as compared to the left FG and IOG (e.g., Rossion et al. 2012; Zhen et al. 2015), while a strong left lateralization for word-selectivity is found in most studies (Cohen et al. 2000; Martin et al. 2015; Wandell 2011). Consistent with the view of dissociated circuitry, we found that a large proportion of the intracerebral contacts responded *only* to faces or *only* to written words and spatial dissociations were found even within core regions of face- and word-processing (FG + sulci and IOG). While there was disproportionately more face (~ 50%) than word (~ 18%) category-selective contacts, this is expected based on previous reports showing a wide distribution of face-selective contacts in *both hemispheres* (Jonas et al. 2016), whereas word-selective contacts are distributed more focally in the left hemisphere only (Lochy et al. 2018).

Do category-selective overlap contacts record different processes than the above-mentioned dissociated contacts? On the one hand, dense clustering of face-selective and word-selective sources in a focal region could reveal itself as overlap on individual iEEG contacts, but still originate from distinct intermingled structures. In this view, there is relatively fewer word contacts (18%) than overlap contacts (30%), because most of the word-selective responses are captured in the overlap contacts, due to word-selective areas containing the intermingled but distinct face-selective structures. On the other hand, the overlap between face- and word-selective responses could reflect shared functional processes (e.g., demand for central-view representations) for these two categories in the same underlying structures (Hasson et al. 2002; Behrmann and Plaut 2013; Plaut and Behrmann 2011). Here, we provide several pieces of evidence suggesting that the face-selective and word-selective amplitudes recorded on the overlap contacts do not reflect shared functional processes in the same microcircuitry.

First, a shared-representational account would predict that face-selective responses would overlap more with word-selective responses than with a control category that does not share the same representational demands. Contrary to this prediction, face- and house-selective contacts showed at least a similar amount of overlap as to face- and word-selective contacts, despite their dissociated neural processes (see Hagen et al. 2020 for a complete report on face and house dissociations in the VOTC). Second, we found no evidence for a positive correlation between face- and word-selective responses in the overlap contacts. This contrasts with the strong between-category correlations of *general visual* responses, which were isolated at a different frequency from the *selective* responses. This latter observation may explain the comorbidity of face and written word recognition difficulties following VOTC damage in terms of a general deficit (either low-level or at a general shape recognition level; Farah 1991; Behrmann and Plaut 2014a, b; Roberts et al. 2015; Rice et al. 2021) and the overlap of activity emphasized in some neuroimaging studies (e.g., Nestor et al. 2012). Moreover, it emphasizes the importance of isolating category-selective responses, by parsing out nonspecific visual responses, when examining functional dissociations. Finally, the dissociated response magnitude in the face- and word-selective amplitudes in overlap contacts in the FG + sulci, which were situated in between face and word contacts on the Talairach X axis, is consistent with these signals arising from distinct neural microcircuits.

We note that the existence of dissociated selective circuitry to faces and written words within the same cortical regions is still consistent with the view that, in line with the general principles of neuronal cortical group selection (Edelman and Finkel 1985; Edelman 1987), these categories may compete for neural representation in the VOTC (Ellis 1983; Allison et al. 2002; Dehaene et al. 2010). For instance, fMRI studies have reported that enhancements of neural activity in the left VWFA with literacy co-occur with small decreases in activation in the same location to faces (Dehaene et al. 2010; Centanni et al. 2018; but see Hervais-Adelman et al. 2019). Moreover, a 3-year longitudinal examination of the functional reorganization in a 6-year-old patient who underwent surgical removal of the right VOTC, showed a protracted increase in both face and written word-selectivity in the left FG and occipito-temporal sulcus (OTS; Liu et al. 2018). Importantly, however, increases of face-selectivity were largely driven by the recruitment of non-selective voxels, suggesting that the competition between faces and words occurs essentially for non-selective neuronal populations (see also Dehaene-Lambertz et al. 2018).

Why do written words selectively recruit populations of neurons in the vicinity of cortical regions also showing strong face-selectivity? One possibility is that lower level foveal retinotopic areas disproportionately project to these higher level areas, and that these areas (e.g., FG + sulci, IOG) are sensitive to the regularity of behaviorally relevant stimuli patterns (Levy et al. 2001; Hasson et al. 2002; Malach et al. 2002; for a discussion on cortical sensitivity to positional regularities, see Kaiser et al. 2019). In a similar vein, higher level visual cortical areas could be strongly connected to downstream larger scale language and social cognitive networks (Peelen and Downing 2017; Persichetti et al. 2021). For example, the emergence of the location of the VWFA as a result of learning to read can be predicted by the structural connectivity of that region and downstream language regions as measured prior to learning (Saygin et al. 2016; for FFA prediction by structural downstream connectivity see, Saygin et al. 2012). An alternative explanation is that these areas contain shared processes crucial for encoding both face and written words, such as the encoding of larger patterns of features (Behrmann and Plaut 2013; for recent discussion, see Op de Beeck et al. 2019). Thus, at a large-scale level, word-selectivity could emerge in a neural system that originally developed mainly for face recognition and that has functional properties that could extend to the word domain. However, there are also important functional differences between faces and words. For instance, faces undergo little or no part decomposition in their representation (i.e., it is “holistic”, or without a category-selective representation of isolated features; Farah 1991; Tanaka and Farah 1993; Rossion 2013). In contrast, even if written words in adults are also processed holistically (Reicher 1969; Farah 1991; Pelli and Tillman 2007), they must also be represented in terms of their individual features (i.e., letters), which are associated to distinct sounds and typically learned independently during reading acquisition. Irrespective of the underlying factors, the current work suggests that within the VOTC of a mature brain, face- and word-selective responses end up in close proximity but nevertheless in clearly spatially dissociated neuro-functional circuitry.

The present study took advantage of the high spatial resolution of depth electrodes coupled with a sensitive frequency-tagging approach that allowed for objective quantification and isolation of selective responses to faces and written words. Collectively, our findings are consistent with the view that face and word recognition in the VOTC is supported by distinct neural populations. However, given the extent of interconnectivity in the nervous system (Sherrington 1906; Bassett and Sporns 2017), future research should examine the connections between different specialized circuitry, using for example in vivo cortico-cortical evoked potentials (Matsumoto et al. 2004), as they could potentially modulate (e.g., inhibit) each other. Indeed, ECoG recordings, although limited in its amount of data (four patients), suggest a mechanism for face-selective circuitry to modulate word-selective circuitry, but not vice versa (Matsuo et al. 2015; see also Allison et al. 2002). Microstimulation of face, word, and overlap contacts could be used to assess whether stimulation in face contacts affect overlap contacts, but not word contacts (and vice versa for words). Finally, an open question that deserves further attention is whether dissociated neural circuitry is specific to highly specialized skills acquired relatively early in life—such as face categorization, reading, and spatial navigation—or whether specialization in any visual domain even in later stages of life ultimately leads to specialized and dissociated circuitry. Indeed, dissociation of neural circuitry could be a fundamental neural organizing principle of the brain for organizing quick and accurate stimulus–response mappings.

## Supplementary Information

Below is the link to the electronic supplementary material.

Supplementary file1 (DOCX 5186 KB)

Supplementary file2 (ZIP 3586 KB)

## Data Availability

Data/material will be made available upon request and will be shared in the Dryad Digital Repository upon acceptance.

## Code availability

Code will be made available upon request and will be shared in the Dryad Digital Repository upon acceptance.
