# Supplementary material for: Dissociated face- and word-selective intracerebral responses in the human ventral occipito-temporal cortex
Source: Brain Struct Funct. 2021 Aug 9;226(9):3031–49. doi: 10.1007/s00429-021-02350-4 (PMC8541991; doi:10.1007/s00429-021-02350-4)
Supplement: Supplementary file 1 — Supplementary file1 (DOCX 5186 KB) [file 429_2021_2350_MOESM1_ESM.docx]

**Supplementary Information for**

**Dissociated face- and word-selective intracerebral responses in the human ventral occipito-temporal cortex**

Simen Hagen^1,2^, Aliette Lochy^3^, Corentin Jacques^4^, Louis Maillard^1,5^, Sophie Colnat-Coulbois^1,6^, Jacques Jonas^1,5^, Bruno Rossion^1,4,5^

1 Université de Lorraine, CNRS, CRAN, F-54000 Nancy, France

2 Donders Institute for Brain, Cognition and Behaviour, Radboud University, 6525 HR Nijmegen, The Netherlands

3 Cognitive Science and Assessment Institute, University of Luxembourg, 365 Esch-sur-Alzette, Luxembourg

4 Psychological Sciences Research Institute and Institute of Neuroscience, UCLouvain, 1348 Louvain-La-Neuve, Belgium

5 Université de Lorraine, CHRU-Nancy, Service de Neurologie, F-54000 Nancy, France

6 Université de Lorraine, CHRU-Nancy, Service de Neurochirurgie, F-54000 Nancy France

**Corresponding Author:**

Bruno Rossion

CRAN UMR 7039, CNRS - Université de Lorraine,

Pavillon Krug, Hôpital Central, CHRU Nancy

29 Avenue du Maréchal de Lattre de Tassigny

54035 NANCY

Tel: +33 (0) 3 83 85 80 53

E-mail: bruno.rossion@univ-lorraine.fr

*Complementary Analysis with Artifact Rejection*. In addition to the analyses reported in the main text, an amplitude quantification analysis after a step of SEEG artifact rejection was performed. For each patient’s data, the following steps were performed: the 63-s sequences were segmented in epochs of duration of one cycle of 1.2 Hz for faces (i.e., 833 ms) and 2 Hz for words (i.e., 500 ms); epochs from separate sequences were pooled together; epochs in which signal amplitude at any time point was above or below 3 times the across-epoch SD were rejected (the mean percentage of rejected epochs across participants was 23.69% ± 5.59% for faces and 15.82% ± 3.88% for words); remaining epochs were averaged together; and a FFT was performed on these averaged epochs (frequency resolution of 1.2 Hz for faces and 2 Hz for words). The face-selective and word-selective visual responses at the face, word and face-word-overlap contacts identified by our main analysis were quantified by using the same methodology.


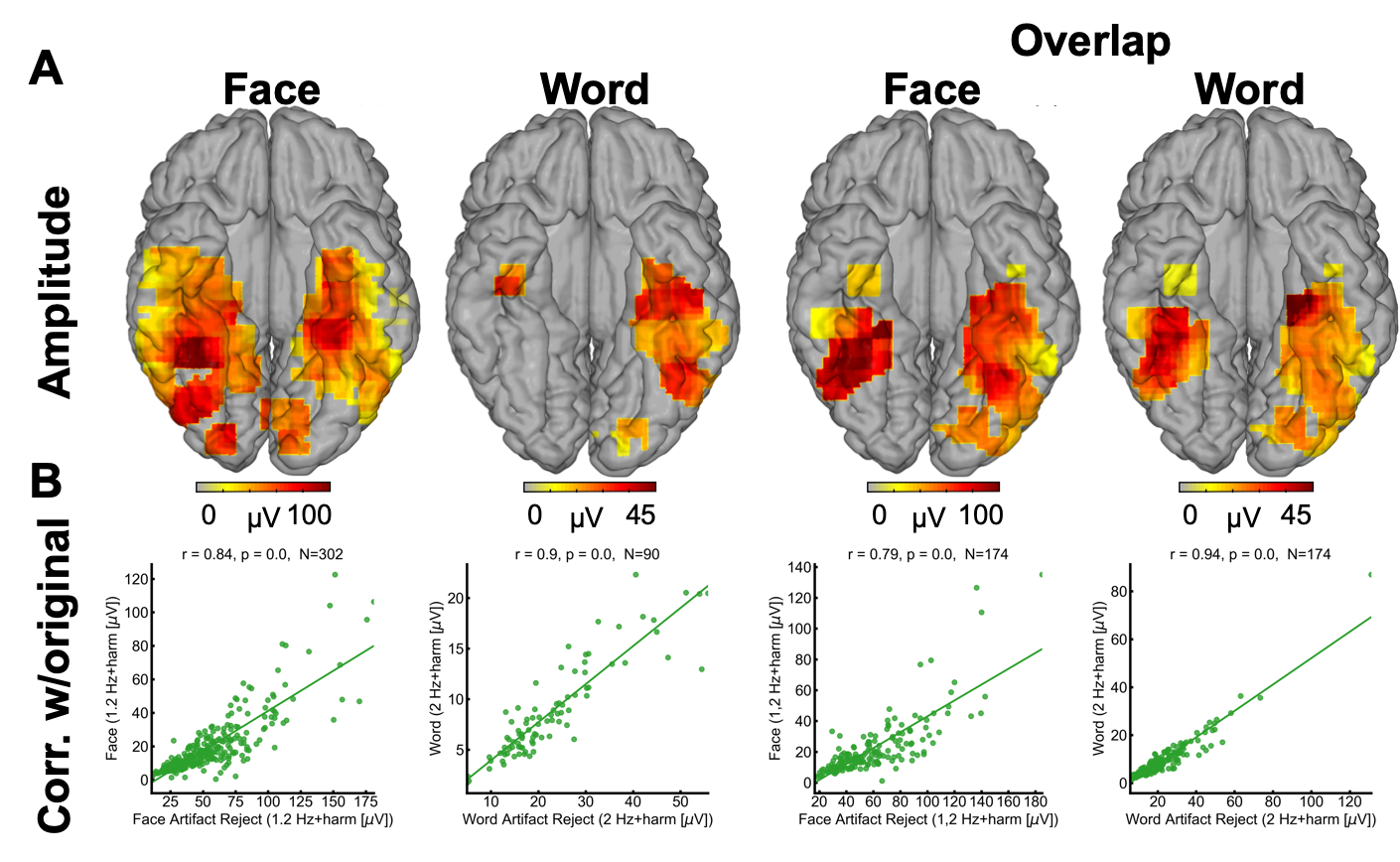


**Figure S1.** *Selective amplitudes after amplitude rejection. A.* *Maps of the local mean selective response amplitudes for faces and words (average amplitude in the significant contacts) for each contact type (face, word, overlap). B. Correlation between the amplitudes before and after artefact rejection.*

*Control analysis for correlations with equal number of harmonics*. In the Figure below, note that the 3^rd^ column shows the original analysis, while column 1 and 2 show the data using the same number of harmonics, 4 and 12 harmonics, respectively. Also note that in the analysis, we removed extreme outliers that when included “artificially” lowered the correlations (max removed in each comparison, n = 4).


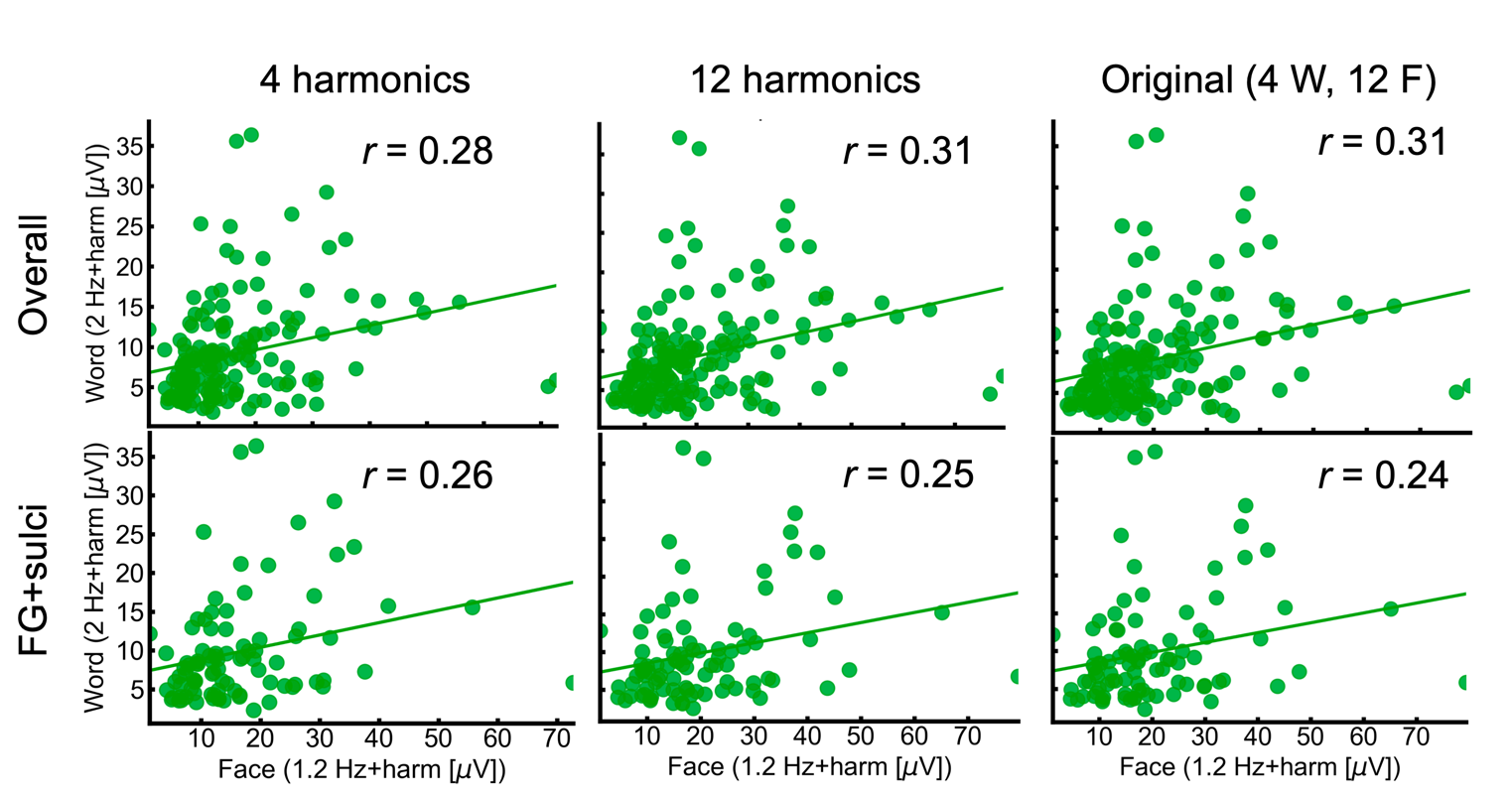


*Figure S2. The low between-category correlation was not affected by the unequal number of summed harmonics for faces and words (compare column 1 and 2 with column 3). Columns 1 and 2 show correlations computed by summing respectively 4 and 12 harmonics of face or word baseline corrected amplitudes. Column 3 shows correlations as in the main manuscript, i.e. computed using different numbers of harmonics for faces and words.*

*Correlation analysis for overlap contacts.* As shown in Figure 8A in the main text, in the face-word-overlap contacts, correlation between the face- and word-selective amplitudes in the left and right hemispheres was not significantly higher than 0, despite a disproportionately strong correlation for within-category amplitudes in both hemispheres. Moreover, a similar pattern was observed for faces and houses in face-house-overlap contacts (Left: face-word: *r* = 0.12, *p* = 0.159, 95% CI = [-0.05, 0.29]; face-face: *r* = 0.91; *p* < 0.001, 95% CI = [0.87, 0.93]; word-word: *r* = 0.72; *p* < 0.001, 95% CI = [0.63, 0.79]; face-house: *r* = 0.11; *p* = 0.235, 95% CI = [-0.07, 0.28]; house-house: *r* = 0.75, *p* < 0.001, 95% CI = [0.66, 0.82]; face-face vs. face-word: *r_diff_* = 0.79; *p* < 0.001; word-word vs. face-word: *r_diff_* = 0.6; *p* < 0.001; house-house vs. face-houses: *r_diff_* = 0.64; *p* < 0.001. Right: face-word: *r* = 0.28; *p* = 0.096, 95% CI = [-0.02, 0.54]; face-face: *r* = 0.89; *p < 0.001* , 95% CI = [0.81, 0.94]; word-word: *r* = 0.69; *p* < 0.001, 95% CI = [0.49, 0.82]; face-house: *r* = 0.21, *p* = 0.067, 95% CI = [0.01, 0.4]; house-house: *r* = 0.58; *p* < 0.001, 95% CI = [0.43, 0.71]; face-face vs. face-word: *r_diff_* = 0.61; *p* = 0.057; word-word vs. face-word: *r_diff_* = 0.41; *p* = 0.012; house-house vs. face-houses: *r_diff_* = 0.37; *p* = 0.039).

In contrast to the selective discrimination responses, for both the face-word-overlap and the face-house-overlap contacts, for the general visual responses there were strong within-condition and between-condition correlations in both hemispheres, indicating that the lack of correlations in the selective discrimination responses cannot be attributed to different levels of noise or attention across conditions, since both the general visual responses and the discrimination responses were measured concurrently within each contact (Figure 8; Left: face-face: *r* = 0.96; *p* < 0.001, 95% CI = [0.95, 0.97]; word-word: *r* = 0.78; *p* < 0.001, 95% CI = [0.71, 0.84]; house-house: *r* = 0.98, *p* < 0.001, 95% CI = [0.97, 0.98]; face-word: *r* = 0.53; *p* < 0.001, 95% CI = [0.4, 0.65]; face-house: *r* = 0.95, *p* < 0.001, 95% CI = [0.93, 0.97]; Right: face-face: *r* = 0.99; *p* < 0.001, 95% CI = [0.97, 0.99]; word-word: *r* = 0.98; *p* < 0.001, 95% CI = [0.97, 0.99]; house-house: *r* = 0.98, *p* < 0.001, 95% CI = [0.97, 0.99]; face-word: *r* = 0.62; *p* < 0.001, 95% CI = [0.4, 0.78]; face-house: *r* = 0.97, *p* < 0.001, 95% CI = [0.95, 0.98]).
 Finally, similar patterns were observed when considering electrode contacts only within the FG+sulci, both for the discrimination responses and the general visual responses (Figure 8B) (Discrimination responses: Left: face-word: *r* = 0.1, *p* = 0.285, 95% CI = [-0.13, 0.32]; face-face: *r* = 0.89, *p* < 0.001; 95% CI = [0.82, 0.93]; word-word: *r* = 0.77, *p* = 0.001; 95% CI = [0.66, 0.85]; face-house: *r* = 0.26, *p* = 0.047, 95% CI = [0.02, 0.48]; house-house: *r* = 0.83, *p* < 0.001, 95% CI = [0.74, 0.9]; face-face vs. face-word: *r_diff_* = 0.79, *p* = 0.007; word-word vs. face-word: *r_diff_* = 0.67, *p* = 0.005; house-house vs. face-house: *r_diff_* = 0.57; *p* < 0.001**.** Right: face-word: *r* = 0.18, *p* = 0.407, 95% CI = [-0.28, 0.58]; face-face: *r* = 0.90; *p* < 0.001; 95% CI = [0.77, 0.96]; word-word: *r* = 0.85, *p* < 0.001, 95% CI = [0.65, 0.94]; face-house: *r* = 0.2, *p* = 0.170, 95% CI = [-0.07, 0.43]; house-house: *r* = 0.46, *p* = 0.002, 95% CI = [0.21, 0.65]; face-face vs. face-word: *r_diff_* = 0.72, *p* = 0.156; word-word vs. face-word: *rdiff* = 0.67; *p* = 0.004; house-house vs. face-house: *r_diff_* = 0.26, *p* = 0.139). General visual responses: Left: face-face: *r* = 0.92, *p* < 0.001, 95% CI = [0.88, 0.95]; word-word: *r* = 0.49, *p* < 0.001, 95% CI = [0.3, 0.65]; face-word: *r* = 0.47, *p* < 0.001, 95% CI = [0.27, 0.63]; house-house: *r* = 0.95, *p* < 0.001, 95% CI = [0.92, 0.97]; face-house: *r* = 0.9, *p* < 0.001, 95% CI = [0.84, 0.94]. Right: face-face: *r* = 0.99, *p* < 0.001, 95% CI = [0.96, 0.99]; word-word: *r* = 0.89, *p* < 0.001, 95% CI = [0.74, 0.96]; face-word: *r* = 0.6, *p* = 0.011, 95% CI = [0.21, 0.82]; house-house: *r* = 0.96, *p* < 0.001, 95% CI = [0.92, 0.97]; face-house: *r* = 0.93, *p* < 0.001, 95% CI = [0.87, 0.96]).
